# A Review on Direct Air Capture of Carbon Dioxide: Sorbent Materials, Process Engineering, Industrial Scale-Up, and Future Perspectives

**DOI:** 10.1021/acs.energyfuels.6c01174

**Published:** 2026-06-29

**Authors:** Nimarta Kaur, Ahmad Al-Bodour, Santiago Aparicio, Mert Atilhan

**Affiliations:** † Chemical and Paper Engineering Department, 4175 Western Michigan University, Kalamazoo, Michigan 49008-5462, United States; ‡ Department of Chemistry, 16725 University of Burgos, Burgos 09001, Spain

## Abstract

The relentless accumulation of anthropogenic greenhouse gases has driven atmospheric carbon dioxide concentrations to approximately 426 ppm, necessitating the aggressive deployment of negative-emission technologies to achieve net zero by 2050. Direct air capture (DAC) offers a scalable, location-independent approach to atmospheric carbon removal; however, it is fundamentally constrained by the significant thermodynamic barriers associated with capturing CO_2_ from ultradilute ambient conditions, requiring minimum thermodynamic energy inputs substantially higher than those for postcombustion point sources. This comprehensive review critically examines the technological landscape of DAC, focusing on the interdependent triad of sorbent material design, contactor engineering, and regeneration thermodynamics. We evaluate the fundamental boundaries of adsorption, emphasizing that an optimal adsorption enthalpy and isosteric heat of adsorption must balance the high CO_2_ uptake capacity with the energetic penalties of sorbent regeneration. A systematic, comparative analysis of state-of-the-art sorbents is presented, encompassing mesoporous silicas, zeolites, carbon-based materials (CBMs), metal–organic frameworks (MOFs), porous organic polymers (POPs), and polymeric membranes. Special attention is devoted to surface functionalization strategies, particularly amine grafting and impregnation, which transition capture mechanisms from physisorption to chemisorption to enhance selectivity under ambient moisture and low partial pressures. Furthermore, we assess the operational merits of various reactor configurations, including gas–solid, gas–liquid, and membrane contactors, alongside regeneration cycles such as temperature, vacuum, pressure, and moisture swing adsorption. Finally, the review bridges fundamental materials science with industrial application by chronicling the scale-up milestones of pioneering entities and providing a strategic roadmap for advancing DAC technology readiness levels toward global deployment.

## Graphical abstract

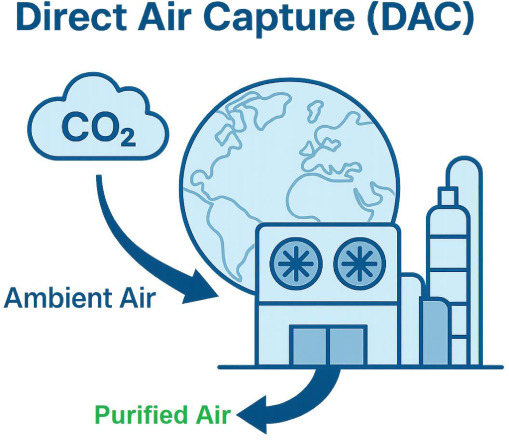

## 1 Introduction

The familiar term “Global Warming”, as heard in the broadcast, should be taken into thoughtful attention as it has reached a level of crisis that is impractical to be overlooked. This climate crisis must be well thought-out for the sake of the forthcoming generation. The recent assessment report (AR6) from the Intergovernmental Panel on Climate Change (IPCC) states that the surface temperature in the last 20 years (2001–2020) has increased by 0.99 °C in comparison to the years 1850–1900. These years were selected to compare that, within just a 20 year span (2001–2020), temperatures have risen at an alarming rate compared to the 50 year span (1850–1950). It was further discussed that the primary reason for this is human activities, particularly the emission of greenhouse gases.^1^ Greenhouse gases consist mainly of carbon dioxide (CO_2_), methane (CH_4_), nitrous oxide (N_2_O), and fluorinated gases which are artificially synthesized. Among these gases, CO_2_ is the primary greenhouse gas accumulating in the atmosphere, and this subsequently contributes to global warming. While the industrial revolution is positively accepted, its exceptional activities do parallelly contribute to increased consumption of fossil-based fuels. This has caused the amount of anthropogenic CO_2_ in the atmosphere to increase by 1% per year over the course of the past decade.^2–4^ CO_2_ alone accounts for 60% of the total contribution to global warming.^5,6^ These trapped CO_2_ remains in the atmosphere for thousands of years creating the phenomenon of “Thickening the Earth’s atmospheric blanket”, hence making the planet warmer.^7^ The atmospheric CO_2_ levels have risen to nearly 426 ppm (parts per million) today, about 50% higher than before the industrial revolution (280 ppm), according to the Mauna Loa Observatory in Hawaii, which has the longest continuous record of direct atmospheric CO_2_ measurements.^8,9^ This climate predicament led to the “Paris Agreement” during the 21st Conference of the Parties, an international treaty aimed at stabilizing greenhouse gas concentrations in the atmosphere to prevent detrimental climate change.^10^

Carbon capture and storage (CCS) processes play an important role in meeting the global warming target. Among the many technologies within CCS, direct air capture (DAC) continues to be studied and is an important component for lowering the carbon footprint. Even though the DAC approach is debated in terms of cost and performance compared to the conventional method of CCS, it may well be a complementary deployment to address climate change and, hence, could act as an “insurance” against CO_2_ accumulation in the air. Furthermore, air capture may serve as an excellent alternative to entrap emissions from mobile, dispersed sources originating from ground, ocean, and air transport.^11,12^

DAC, introduced by Professor Klaus Lackner from Arizona State University in 1999, demonstrated that this method can be one of the technical approaches for capturing CO_2_ from the air. The implementation of DAC can be rooted to a deeper understanding of the physical nature of gas behaviors under certain temperatures and pressures and their capability to bind to a solid. The DAC methodology is able to function around the world as one of the approaches today to capture CO_2_, owing to the discovery and evolution of “sorption” over the years.^13,14^ Figure 1 shows a comparison between DAC technology and point-source CCS.

**Figure 1:**
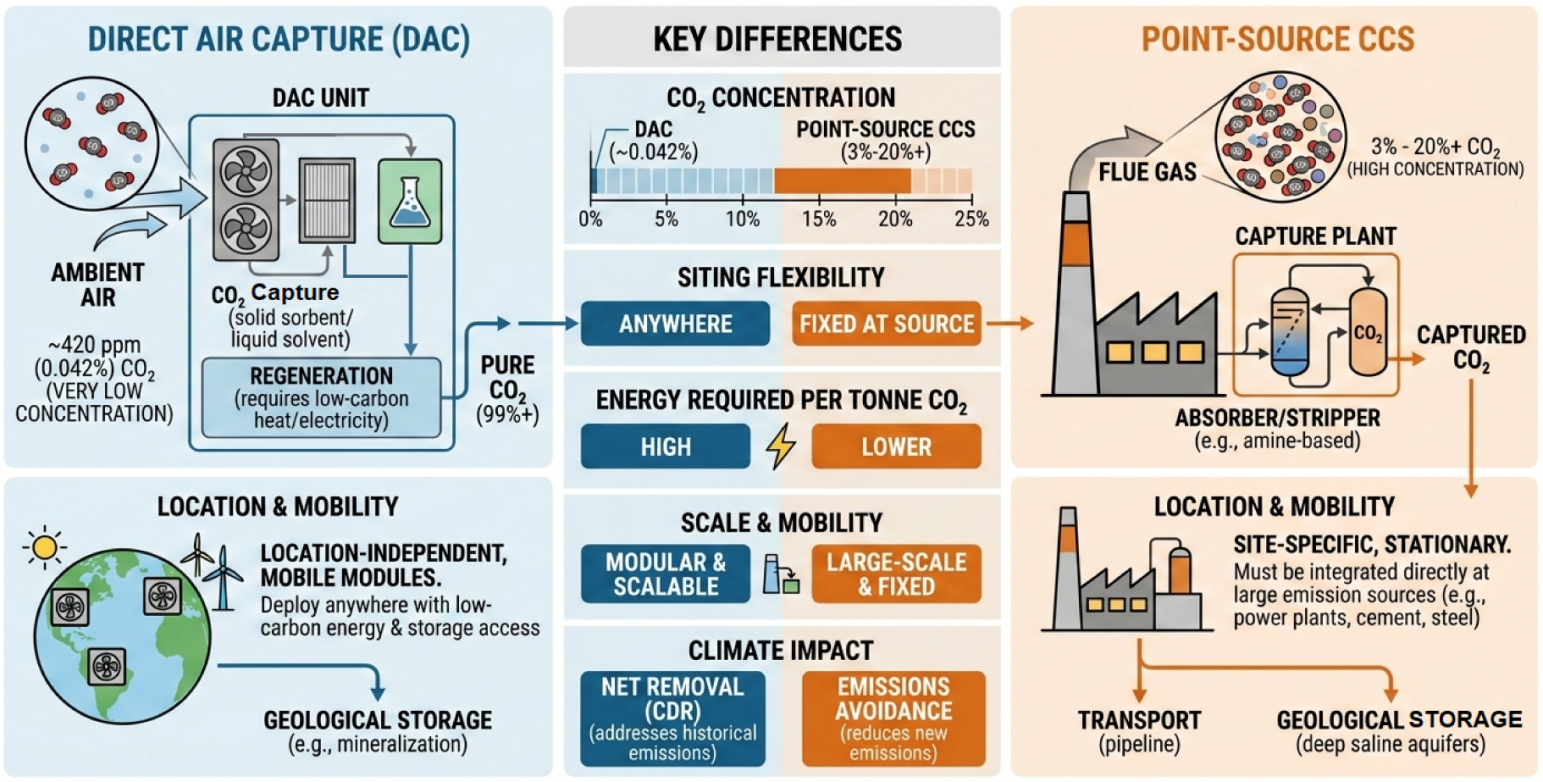
Two Complementary Climate Tools: DAC for permanent carbon removal and CCS for targeted emissions mitigation, informing balanced policy portfolios for net-zero strategies.^11,12,15–17^

As opposed to the conventional method involving high temperatures for capturing CO_2_, DAC is conducted at atmospheric pressure and temperature to combat climate change. CO_2_ removal from coal-firing plants and other flue gas-generating plants is ongoing and important; however, the alertness and necessity of removing the CO_2_ that has already been added to the air are correspondingly imperative to cool down earth’s reflectivity.^15,16^ The range of DAC is in accordance with the National Institute of Standards and Technology (NIST), and it uses a temperature of 20 °C (293.15 K, 68 °F) and an absolute pressure of 1 atm (14.696 psi, 101.325 kPa). The application extraneous to climate change was first introduced in the 1930s, cryogenically separating air from plants, and subsequently developed for closed ecosystems such as space stations or submarines. Modern space shuttles are equipped with the same analogy that can be regenerated to provide habitable conditions for the crewmembers on board.^18–20^ For climate purposes, DAC was theoretically proposed by the founders of Climeworks in 2009, and they initiated the first operational-scale plant in Orca, Ireland.^21^ As of now, 27 DAC plants are fully operational, capturing nearly 0.01 Mt of CO_2_ per year, and 130 DAC facilities are in various stages of development. If these 130 DAC plants are developed by 2030, it would be feasible to encapsulate 65 Mt of CO_2_ per year, facilitating the notion of the Net Zero Emissions scenario by 2050.^22,23^

All in all, DAC can be considered in the same line as bioenergy for capture and storage (BECCS). This method utilizes biomass such as plants and crop residues to produce energy either by thermochemical or biological processes.^17^ To compare these two capture methods, DAC can be defined as the “man-made shrub” which can be debated whether it is important to develop or not. Nevertheless, the term “not optimal but serviceable” allows for more reasoning in developing more raw materials, resources, and technology to overcome global climate warming. This paper will converse different types of adsorbent materials based on their performance to facilitate CO_2_ capture directly from the air.

In recent years, a growing number of review articles have addressed various aspects of DAC. A recently published review paper was by Filahi et al. (2025). They reviewed the DAC material development such as zeolites, amine-modified mesoporous solids, and other aspects in terms of greenhouse cultivation facilities.^24^ Chowdhury et al.’s (2023) review paper was focused on the DAC pilot scale projects and industrial scale deployments.^25^ Li et al. (2025) published a mini-review on DAC and direct ocean capture (DOC) focusing more on the electrochemical routes.^26^ Other reviews are more focused on equipment and process design; for example, Kourou et al. focused on contactor designs in addition to intensification strategies.^27^ Also, Hu et al.’s (2025) review conducted a bibliometric analysis of the research papers in the DAC field in addition to the focus on the process systems engineering aspects of the DAC.^28^

Despite this extensive literature, a clear gap remains in the systematic integration of adsorption thermodynamics, experimental measurement practices, and DAC-relevant process operation within a single framework. Unlike recent reviews that emphasize either materials development, process design, or deployment individually, this work explicitly links sorbent-level adsorption behavior with air contactor design, regeneration energetics, and industrial scale-up considerations. This review is particularly timely as DAC technologies transition from pilot-scale studies to early commercial deployment, where inconsistencies between laboratory performance metrics and full-scale energy requirements are increasingly evident. While these contributions have significantly advanced the field, most studies focus on either sorbent performance or process configurations in isolation with limited integration of adsorption thermodynamics, measurement methodologies, and practical DAC system design. In particular, important topics such as the distinction between excess and absolute adsorption, adsorption mechanisms operative under ultradilute CO_2_ conditions, and the coupling between material properties and air contactor and regeneration strategies are seldom addressed within a unified framework. This review provides a systematic and comprehensive synthesis that connects adsorption fundamentals, major DAC material classes, and process configurations, including air contactor designs and regeneration methods. In addition, recent developments in industrial DAC technologies are contextualized alongside material-level performance metrics and energetic considerations. Given the rapid acceleration of DAC deployment and the strong dependence of feasibility on sorption and regeneration energetics, such an integrated and structured perspective is both timely and necessary to guide future research and development.

## 2 Fundamentals of CO_2_ Capture

As a rule, two stages are involved in the DAC process of CO_2_: adsorption and desorption. The first stage of adsorption involves ambient air passing through a selective adsorbent, capturing CO_2_ molecules and allowing the purified air to pass through. The process reaches equilibrium, which is associated with the final amount of CO_2_ captured. During the desorption stage, the captured CO_2_ is released from the sorbent and collected in order to be utilized or kept in storage.^29^

### 2.1 Thermodynamics of Adsorption

CO_2_ apprehension by an adsorbent must be accounted for on all sides of thermodynamic behaviors: first being the adsorption enthalpy Δ*H*_ads_, which depicts the strength of the surface of the host when binding with the arrival of the CO_2_ molecule from the air; second being the isosteric heat of adsorption *Q*_st_; and third being the pore volume. Enthalpy Δ*H*_ads_ must be more than the range of −50 to −60 kJ mol^–1^ in order to have a healthy uptake of CO_2_ under DAC conditions. The negative value of *Q*_st_ which equals the positive Δ*H*_ads_, i.e., Δ*H*_ads_ = −*Q*_st_, accounts the same manner of how it must be higher than 50 to 60 kJ mol^–1^. This reflects thermodynamically how an adsorbent is feasible in capturing CO_2_ from the air. Contrarily, −*Q*_st_ must not exceed values above 60 kJ mol^–1^ because this would lead to higher energy requirements to reactivate the sorbent back to its initial state. Third is the pore volume which dictates the maximum adsorption capacity and involves the diffusion of the CO_2_ molecule and the binding governed by the pore’s textural size and properties.^30,31^ It is evident that there is a thermodynamic impediment to capturing CO_2_ from the air, which has a partial pressure 300 times lower than the partial pressure of CO_2_ in flue gas. The mathematical amount of energy required to separate these gases is proportional to the log of the partial pressure, which is vastly divergent. Hence, at a minimum and thermodynamically, capturing CO_2_ from ambient air requires twice as much energy in contrast to capturing CO_2_ from postcombustion exhaust.^16,32^ The heat of adsorption can be precalculated by implementing the Clausius–Clapeyron equation. These calculations which involve two isotherms can determine the equilibrium constant, providing a deeper understanding of the thermal effect of CO_2_ adsorbed on the sorbates.^33^ In DAC, CO_2_ separation occurs under ultradilute conditions (≈400–425 ppm),^34,35^ where adsorption is governed by narrow thermodynamic margins. Adsorption thermodynamics directly determine the minimum achievable capture efficiency, selectivity, and regeneration energy for DAC sorbents. In particular, the enthalpy and Gibbs free energy of adsorption control the trade-off between CO_2_ uptake at low partial pressure and the energy penalty required for sorbent regeneration. Sorbents with weak physisorption typically exhibit insufficient affinity for CO_2_ under DAC conditions, while excessively strong binding leads to prohibitively high thermal or vacuum regeneration demands. Therefore, understanding thermodynamic constraints is essential for evaluating sorbent feasibility and for interpreting performance metrics discussed in subsequent sections on DAC materials and regeneration strategies. Figure 2 illustrates adsorption strength and regeneration energy for physisorption and chemisorption.

**Figure 2:**
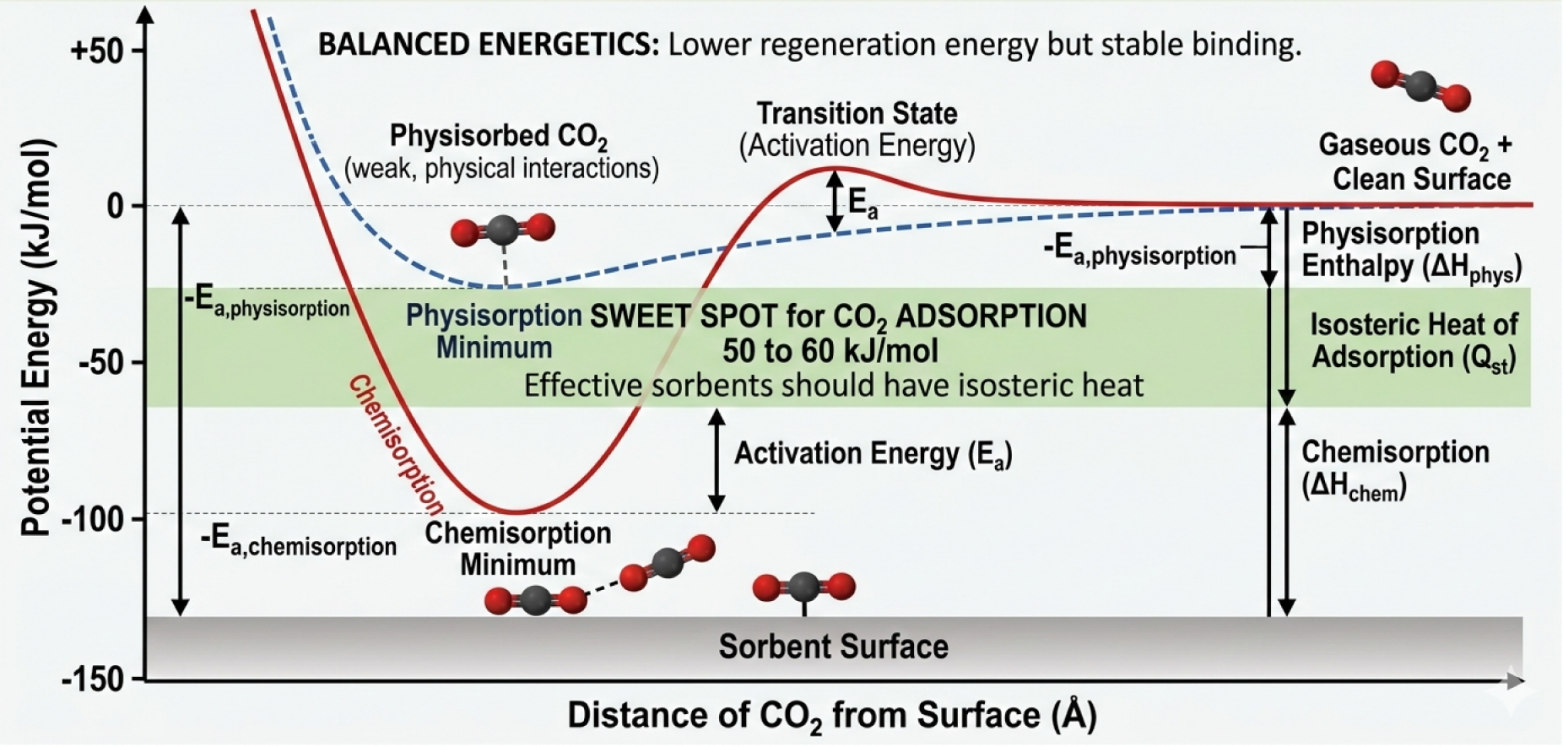
Potential-energy profiles for CO_2_ surface interactions with solid sorbents: Comparison of the physisorption pathway (dashed blue line) and the chemisorption pathway (solid red line) relative to the gaseous CO_2_ state.^30,31,33,36,37^

### 2.2 Physisorption vs Chemisorption

The two mechanisms of CO_2_ sorption are physisorption and chemisorption. Physisorption involves only surface phenomena, interactions between the adsorbate and the CO_2_ molecule, throughan exothermic process that is temperature- and pressure-dependent. Van der Waals forces and electrostatic interactions, including quadrupole–ion and quadrupole–dipole interactions between CO_2_ and the ionic or polar sites of the adsorbent surface, are the primary driving forces governing CO_2_ adsorption in porous solid adsorbents.^37,38^ Chemisorption involves an ionic or covalent bond between the functional sites of the modified adsorbents and the CO_2_, and it depends on acid–base neutralization reactions.^38,39^ From this, it seems apparent that physisorption which does not involve any covalent or ionic bond exchange requires less energy for adsorbent regeneration. In the context of DAC, the distinction between physisorption and chemisorption has direct implications for sorbent screening and process selection, as discussed in subsequent sections. Because DAC operates at ultralow CO_2_ partial pressures and in humid environments, physisorption-dominated materials often exhibit limited working capacities and strong sensitivity to water vapor, whereas chemisorption-based materials typically provide higher selectivity under ambient conditions. However, the stronger binding associated with chemisorption results in higher regeneration temperatures or longer desorption times, directly affecting energy consumption and cyclic stability. Consequently, the balance between physisorption and chemisorption mechanisms plays a central role in determining the suitability of different material classes for DAC system applications.

### 2.3 Measurement Considerations (Excess vs Absolute)

It is crucial to evaluate different types of adsorbates for air capture since we know that pressure is directly proportional to physisorption. Physisorption at low pressure fills up pores to the actual adsorbed amount of the CO_2_ molecule, which is called excess adsorption. However, with high pressure, this then leads to an absolute adsorption measurement of the CO_2_ molecule. This absolute adsorption allows multilayers of CO_2_ molecules to bind physically to the adsorbate since the pressure alters the gas density.^40,41^ In the DAC domain which involves adsorption at low pressure, the pore volume-filling mechanism is the primary driving force of the adsorption process, with micropores playing a significant role in CO_2_ adsorption.^42^ For DAC-relevant conditions, where adsorption occurs at near-atmospheric pressure and very low CO_2_ concentrations, the distinction between excess and absolute adsorption becomes particularly important when interpreting reported sorbent capacities. Many DAC sorbents exhibit relatively low excess uptake values despite favorable performance under cyclic operation, which can lead to inconsistent comparisons across studies if adsorption metrics are not defined consistently. Misinterpretation of excess adsorption data may therefore result in over- or underestimation of sorbent performance and regeneration efficiency. Clarifying this distinction is essential for linking laboratory adsorption measurements to realistic DAC operating conditions and for enabling meaningful comparisons among the sorbent classes.

## 3 Sorbent Materials for DAC

An important part of the DAC systems is the sorbent. The ideal sorbent for the DAC process must satisfy a set of conditions. Before fulfilling that set of conditions, there are a set of parameters that need to be satisfied: the process of capture must be performed in ambient environments because pressurizing, heating, or cooling a large amount of air is uneconomical, and the energy utilized for the capture process must come from a noncarbon-based source. This is to ensure that there are no more emissions of CO_2_ within the process of capture. After the fulfillment of these requirements, the following list of sorbent characteristics must be fulfilled based on the applications: 1) high selectivity, 2) high capacity, 3) fast transport and kinetic properties, 4) thermal and chemical stability, 5) mechanical properties (solid sorbent), 6) ease of loading (solid sorbent), 7) resistance to fouling, 8) ease of regeneration, and 9) low cost. Even though there is no material that satisfies all these characteristics, continual research work has been conducted to develop various solid- and liquid-based sorbents for the DAC of CO_2_ purposes. Examples include solid-supported amines, alkali and alkaline earth bases, and metal-organic frameworks (MOFs).^35^

The adsorption process, which falls under the category of solid DAC (S-DAC), utilizes the porous surface of the solid material that acts as a filter to trap atmospheric CO_2_. Other being liquid DAC (L-DAC) used as a solvent-based material to absorb CO_2_ into a homogeneous solution. S-DAC is preferred over L-DAC due to the high selectivity of CO_2_ from air, regeneration, and stability, which can be favorable factors for a long operational lifespan.^43,44^

Assortments of CO_2_ capture sorbates directly from air are strategized based on the temperature and pressure of the CO_2_ point source, the partial pressure of CO_2_, its concentration, and volume. In addition, external factors aside from the adsorption process such as impurity and pollutant levels in the air itself, the efficiency of the capture process and the regenerations, the cost involved, and the impact it has on the environment are taken into consideration to determine which type of sorbate is chosen.^42,45–47^ Numerous materials have been extensively explored as sorbents such as activated carbon, MOFs, alkali oxides, zeolites, etc. Generally, sorption materials can be categorized based on the sorption mechanism as physical sorbents and chemical sorbents. Most experimental studies on DAC sorbents report excess adsorption capacities, as these are directly obtained from conventional adsorption measurements, whereas absolute adsorption is less frequently reported or consistently corrected for under DAC-relevant conditions.

### 3.1 Physical Sorbents

Physical sorbents require less energy to release the captured CO_2_ gas during the regeneration stage compared with chemical-based sorbents. This is due to the weaker physical interactions of van der Waals forces between the sorbent and CO_2_.^48^ Usually, physical sorbents are not often reported in the context of DAC applications and are frequently coupled with chemical sorption. The following section is dedicated to reporting the physisorption materials for DAC applications.

#### 3.1.1 Zeolites

Zeolites have been opted lately for DAC due to their good attainment of CO_2_ adsorption at low pressure, and their inherent active metal shows interaction with CO_2_ molecules.^49^ The structure is of a crystalline type, composed of silicon and aluminum, with different physical porosity and geometry. The distinct types of zeolite formation are determined by the disparity in parameters, particularly the ratio of aluminum to silicon.^50^ CO_2_ DAC capture in zeolites is inclined by the size, charge density, and distribution of cations in the porous structure.^51^ Recently, huge research efforts have been concentrated on the synthesis of modified zeolite molecular sieves for the enhancement of their CO_2_ sorption capacity. For example, introducing functional groups or metal ions like lithium, potassium, sodium, etc. can substantially enhance the zeolite’s adsorption capacity for CO_2_ gas.^52^ Moreover, the employment of various methods of synthesis such as solid-phase synthesis and hydrothermal synthesis showed effective adjustments to the structural properties of zeolites for enhanced CO_2_ adsorption performance.^53^ In CO_2_ gas capture, zeolite material performance is strongly connected to their surface functional groups, particularly metal cations (Ca^2+^, Na^+^, K^+^) and hydroxyl groups (OH^–1^). Hydroxyl groups improve the affinity of the material for CO_2_ via hydrogen bonding, while metal cations facilitate selective CO_2_ capture through electrostatic interactions. Consequently, functional groups’ precise regulation, like using cation exchange or modifying the surface, became a key method for accomplishing CO_2_ capture effectively using zeolites. Table 1 shows several different occurrences of CO_2_ capture under ambient air conditions by different zeolite groupings.

**Table 1:** CO_2_ Adsorption Performance by Zeolites under the Conditions of 1 bar CO_2_ and 25 °C Temperature from Static Equilibrium Isotherms

Sorbent Material	Capacity (mmol/g)	Ref
Zeolite 13X	5.49	^54^
AQSOA Z02A + MOR	1.10	^55^
Zeochem-Z10-02 + Zeolite 13X	3.64	^56^
Zeochem Z4-01 + Zeolite 4A	3.07	
Ca-X	5.20	^57^
Ca-A	5.10	
Hierarchical zeolite-Y	5.40	^58^
Na-CHA	4.70	^59^
K-CHA	2.10	^60^
SSZ-45	1.40	^61^
Na-ZSM 5	2.10	^62^
Li-SSZ-13	5.10	^63^
Na-SSZ-13	4.90	
K-SSZ-13	3.80	
Zeolites X-Li	5.62	^64^
Zeolites X-Na	4.98	
Zeolites X-K	4.44	
Zeolites X-Rb	4.08	
Clinoptilolite-Li	1.20	^65^
Clinoptilolite-Ca	1.00	
Clinoptilolite-Mg	0.80	
13X-Li	6.98	^66^
13X-Na	6.28	
13X-Ca	5.00	^67^
ZK-5-Li	4.90	^68^
SSZ-13-Ni	4.47	^69^
SSZ-13-Co	4.49	
SSZ-13-Zn	4.10	
Nano zeolite-Cu	7.16	^70^
Nano zeolite-Fe	6.23	
SSZ-13-H	3.98	^71^
SSZ-13-Cu	3.75	

#### 3.1.2 Carbon-Based Materials (CBMs)

In the ancient world, activated carbon was used to absorb unpleasant odors, purify water, and as a cure for many diseases. In the 18th century, Carl Wilhelm Scheele, a Swedish–German chemist, experimented on the amount of air adsorbed and desorbed.^14^ CBMs have been studied recently due to their high surface area-to-volume ratio, fast adsorption and desorption kinetics of CO_2_ molecules, and great regeneration.^38^ Activated carbon, originating from biomass and utilizing the process of pyrolysis, has been detailed for ambient air capture. Pyrolysis eans heating biowaste in an inert atmosphere at high temperatures of up to 900 °C to yield a biochar with characteristics similar to charcoal.^72^ Other types of CBMs such as carbon membranes , carbon aerogels, heteroatom doping, metallic doping, and carbon nanotubes (CNTs) have been reported in ambient air CO_2_ capture.^38^ Various methods were explored to prepare modified versions of activated carbon to improve their CO_2_ adsorption capacity and selectivity by introducing functional groups or adjusting the pore structure. For example, the activated carbon modification using amine-based materials exhibited a significant increase in its affinity toward CO_2_, thus enhancing its performance in applied applications.^73^ Ma’s group^74^ exploited the coordination between functional groups and zinc ions in the stems of tobacco to produce zinc gas. This notably improved the volume of the ultramicropores and the capacity for CO_2_ adsorption, achieving values of 209 mg/g and 146 mg/g at 0 and 25 °C, respectively. The produced material exhibited outstanding selectivity for CO_2_ gas against nitrogen and notable benzene adsorption capacity at minimal concentrations, with magnitudes of 97 mg/g and 202 mg/g at 10 and 50 ppm, respectively. In the process of CO_2_ gas capture, functional groups like the amine group (−NH_2_) added to activated carbon improve the affinity of the material toward CO_2_ through surface interaction, remarkably enhancing both sorption capacity and selectivity. This is due to the ability of the amine functional group to interact with CO_2_ gas forming stable carbamates. In this manner, the adsorption performance and selectivity will be significantly improved. As a result, the introduction of these functional groups via chemical modification has become a key strategy to improve the adsorption efficiency of activated carbon materials. Table 2 shows a number of different occurrences of CO_2_ capture under ambient air by the CBM grouping.

**Table 2:** CO_2_ Adsorption Performance by CBMs under the Conditions of 1 bar CO_2_ and 25 °C Temperature from Static Equilibrium Isotherms

Sorbent Material	Modification	Capacity (mmol/g)	Ref
Activated carbon, G-32 H		2.25	^56^
Sorbent-coated carbon fibers	Mesoporous silica (SYLOID C-803) and cellulose acetate (CA) using a roll-to-roll coating system and then impregnated PEI within the coating layer	1.20	^75^
Activated carbon fabric ACC-509210	Potassium hydroxide (99%), potassium bicarbonate (99%), and sodium hydroxide (99%)	0.26	^76^
Amine-impregnated AC from *Jatropha*	Triethanolamine (TEA)	0.58	^77^
Na_2_ CO_3_ supported AC honeycomb	Sodium carbonate grafting	0.75	^78^
Hydrated K_2_CO_3_ on AC honeycomb	Potassium carbonate hydration	0.65	^78^
Activated porous biocarbons	Nitrogen functionalization	0.65	^79^
AC from PET waste	KOH chemical activation	0.72	^80^
Modified porous carbon adsorbents	Activating agent	0.55	^81^
Activated carbons from biomass		0.68	^72^
Graphene oxide	Ethylenediamine (EDA)	1.18	^82^
Graphene oxide	Polyaniline (PANI)	1.31	^83^
Subtropical macroalgae (*Sargassum horneri*)	KOH activation	2.78	^84^
Graphene oxide	Ultraviolet activation	1.83	^85^
Graphene oxide	Binder (glucose), GO framework, trimethylcarbinol	1.73	^86^
Carbon nanotubes	HNO_3_, H_2_SO_4_	0.32	^87^
Carbon nitride frameworks (CNFs)—CNF-1		3.35	^88^
CNF-2		3.05	
Hierarchical porous carbons (HPCs) HPCs-5-900		2.84	^89^
HPCs-5-900		2.25	
AC-600-1		3.67	^90^
AC-600-2		3.64	
AC-600-0.5-H		2.76	
AC-700-1		4.10	
AC-700-2		3.77	
AC-700-0.5-H		4.13	
AC-800-0.5		4.03	
AC-800-1		3.00	
AC-800-2		2.51	
AC-600-0.5-H		2.76	
AC-750-0.5		4.30	
N-doped porous carbons (from water chestnut shell)	NaNH_2_	4.50	^91^
N-enrich porous carbon-NPCs (derived from glucose)		3.20	^92^
N-doped carbon derived from NUT-4		3.70	^93^
N-doped carbon derived from octaphenylcyclotetrasiloxane		3.32	^94^
N-doped carbon derived from polyamine		4.40	^95^
N-doped carbon derived from polyethylenimine		4.92	^96^
N-doped carbon derived from hexamethylenetetramine		4.21	^97^
S-doped carbon derived from heavy coker gas oil		2.60	^98^
N, P codoped carbon derived from triazine polymer		1.52	^99^
*Arundo donax*	Solid KOH	3.60	^100^
*Arundo donax*-acidified	Solid KOH	3.20	^101^
Bee-collected pollen (1:1)	Aqueous KOH	3.38	^102^
Bee-collected pollen (1:1)		3.42	
Black locust		5.05	^103^
Carrot peels		4.18	^104^
Fern leaves		4.12	
Pomegranate peel		4.11	
Celtuce leaves		4.36	^105^
Coconut shell	CO_2_ activation	3.90	^106^
Coffee ground	Aqueous KOH	3.00	^107^
Coffee ground	CO_2_ activation	2.40	
Coffee ground	Aqueous KOH	4.00	^108^
Date sheets	Aqueous KOH	3.65	^109^
Enteromorpha	Aqueous KOH	0.52	^110^
*Sargassum*	Aqueous KOH	1.05	
Granular bamboo	Aqueous KOH	4.50	^111^
Lumpy bracket	Aqueous KOH	4.62	^112^
Nutshell	CO_2_ activation	3.48	^113^
Olive stones		1.98	^114^
Palm kernel shell	CO_2_ activation	2.13	^115^
Palm shells	Aqueous KOH	4.40	^116^
Peanut shell char	Aqueous KOH	4.41	^117^
Rice husk char	CO_2_ activation	3.10	^118^
Rice husk char	Aqueous KOH	3.71	^119^
Teas seed shell	Aqueous KOH	3.15	^120^
Garlic peel	Aqueous KOH	4.22	^121^
Sugar cane bagasse	Aqueous KOH—Urea	4.80	^122^
GO-EDA	Ethylenediamine/GO	1.18	^82^
10UV-GOF	UV-irradiated GO	1.83	^85^
TiO_2_/GO		1.88	^123^
MgO/CaO		2.19	^124^
UiO-66/GO		3.37	^125^
NiO/PCNF		1.11	^126^
CaO loaded charcoal		8.00	^127^
Glucose/graphene-based aerogels (G/GAs)	Hydrothermal reduction and CO_2_ activation	1.73	^86^

#### 3.1.3 Metal–Organic Frameworks (MOFs)

Metal–organic frameworks (MOFs) are porous organic–inorganic crystalline hybrid materials governed by the self-assembly of metal atoms and organic linkers.^128^ The utilization of MOFs has presented characteristic benefits for CO_2_ capture, including adjustable pore geometry, large surface area, structural flexibility, and the ability to act as an excellent host in the world of chemistry to develop different sets of materials.^129–131^ MOFs are considered industrially significant since they possess the capability to develop several tunable physical attributes which is a good strategy to capture CO_2_ from air such as changeable pore size and shape and the fashioning of polar pore surfaces.^129,132,133^ Further modifications incorporate adding open metal sites (OMSs), hydrogen-bonding receptors, appended amine sites, and electrostatic interaction sites.^134–137^ Lately, several researchers focused on the enhancement of the CO_2_ sorption capacity of MOFs via chemical functionalization using the introduction of polar groups like carboxyl and amino groups.^138^ For example, amino-functionalized MOF-5 showed a high selectivity and sorption capacity of CO_2_ at low pressure magnitudes, demonstrating its capability for industrial-scale flue gas purification.^139^ MOFs have extraordinary adsorption capacity of CO_2_ originated from the synergetic impacts of different functional groups within their structures like hydroxyl (−OH), amino (−NH_2_), and carboxyl (−COOH). The chemical interaction between CO_2_ and amino groups particularly boosts the MOFs’ adsorption performance significantly by forming carbamates. In the meantime, hydroxyl and carboxyl groups improve CO_2_ selectivity and affinity further via polar interactions and hydrogen bonding. Additionally, the unsaturated metal sites in MOFs can function as active sites directly and manifestly enhancing the selectivity of CO_2_ sorption. Table 3 shows a number of different occurrences of CO_2_ capture under ambient air by various MOF groupings .

**Table 3:** CO_2_ Adsorption Performance by the Assembly of MOFs under the Conditions of 1 bar CO_2_ and 25 °C Temperature from Static Equilibrium Isotherms^a^

Sorbent Material	Modification	Capacity (mmol/g)	Ref
HKUST-1		0.05	^54^
Mg-MOF-74		0.09	
Mg-IRMOF-74-II	N,N′-Dimethyl ethylenediamine (mmen)	2.00	^138^
Mg_2_(dobdc)-ed	Ethylenediamine (ED)	1.51	^140^
Mg_2_(dobdc)(N_2_H_4_)1.8	Toluene solution of anhydrous N_2_H_4_	3.89	^141^
Mg_2_(dobpdc)-mmen	Dimethylformamide (DMF)	3.00	^142^
Mg_2_(dobpdc)-en	Ethylenediamine (ED)	2.83	^143^
Cr-MIL-101-SO_3_H-TETA		1.12	^144^
NbOFFIVE-1-Ni	Pyrazine, Ni(NO_3_)_2_·6H_2_O, Nb_2_O_5_ and HF	1.30	^145^
NbOFFIVE-1- Ni@PA		1.44	^146^
MIL-101(Cr)-TREN		0.35	^147^
MIL-101(Cr)-PEI-800		1.20	
SIFSIX-3-Ni		2.42	^54^
SIFSIX-3-Cu		1.24	^148^
SIFSIX-3-Zn		0.13	
SIFSIX-3-Ni		0.18	
SIFSIX-2-Cu-i		0.07	
SIFSIX-2-Cu-i	4,4′-Dipyridylacetylene (DPA)	4.60	^149^
SIFSIX-3-Zn	Pyrazine (PYR)	2.40	
SIFSIX-18-Ni-β	3,3′,5,5′-tetramethyl-1H,1′H-4,4′-bipyrazole with NiSiF_6_·6H_2_O	0.96	^150^
TIFSIX-3-Ni	Ni(pyrazine)_2_	1.15	^151^
TIFSIX-3-Co	Co(NO_3_)_2_·6H_2_O, (NH_4_)_2_TiF_6_ and pyrazine	1.05	^152^
GeFSIX-3-Ni		1.07	^153^
GeFSIX-3-Co		0.30	
SIFSIX-3-Cu		1.24	^148^
NbOFFIVE-1-Ni		1.30	^136^
SIFSIX-3-Ni		0.42	^151^
TIFSIX-3-Ni		1.11	
SIFSIX-2-Cu-i		0.07	^149^
[Zn(ZnOH)_4_(bibta)_3_]		2.20	^154^
Zn_5_(OH)_4_(btdd)_3_		1.54	^155^
IITKGP-5a		1.98	^133^
CFA-1-OH(Zn)		2.20	^156^
CFA-1-OH(Ni)		2.70	
UTSA-400 (WO_2_F_4_-1-Ni)		0.93	^157^
TIFSIX-3-Co (ZU-16-Co)		1.05	^152^
UiO-66		0.02	^158^
ZIF-8		0.05	
MIL-101(Cr)		0.02	
Pyridyl-decorated MOF-505		3.20	^159^

a M_2_(dobpdc) (M = Zn (**1**), Mg (**2**); dobpdc^4–^ = 4,4′-dioxido-3,3′-biphenyldicarboxylate).

#### 3.1.4 Porous Organic Polymers (POPs)

POPs is considered a prime candidate for CO_2_ DAC capture since its key factors include permanent porosity, structural tunability, stability, and relatively low cost.^160^ Covalent Triazine Frameworks (CTFs) are one of the classes of POPs first introduced by Professor Arne Thomas back in 2008. These CTFs synthesized through the trimerization of aromatic nitriles are considered for capture due to their conjugated nature, high heteroatom content, and surface areas.^161^ Developments such as cavitands allow CTFs to act as preorganized scaffolds to entrap CO_2_ from ambient air. Multiple cavitands can unite to build CTFs cages, materializing greater capacity and stability to capture CO_2_. Two types of modifications of POPS undertaking catalytic performance are presented: 1) Organocatalyst and 2) Heterogeneous catalyst. Organocatalytic development of POPs was introduced by Yavuz et al., demonstrating the first approach based on a simple polymer featuring pyridyl salicylimines. Imidazolium-based POPs with exceptional catalytic performance to capture CO_2_ as a substrate are also reported in the following table. Metal-ion POPs configurations allow the formation of heterogeneous catalysts for CO_2_ conversion. The metal sites offer high affinity to polarize the CO_2_ molecule converting it into an inert molecule.^160,162^ Table 4 shows a number of different occurrences of CO_2_ capture under ambient air by the POPs grouping.

**Table 4:** CO_2_ Adsorption Performance by the Assembly of POPs under the Conditions of 1 bar CO_2_ and 25 °C Temperature from Static Equilibrium Isotherms

Sorbent Material	Modification	Capacity (mmol/g)	Ref
COP-190H-en	Ethylenediamine (EN)	2.19	^163^
COP-190H-deta	Diethylene triamine (DETA)	1.79	
COP-190H-CN	Sodium cyanide (NaCN)	2.11	
COP-190H-SH	Sodium hydrosulfide (NaHS)	2.28	
TBOSBL1	2,6-Diaminotriptycene (DAT)	2.10	^164^
TBOSBL2		2.60	
TBOSBL3		2.20	
PMOP		3.17	^165^
PFPOP-1		1.20	^166^
PFPOP-2		1.50	
PFPOP-3		1.70	
TAP1	Triptycene amines	1.40	^167^
TAP2		2.30	
TAP3		2.30	
Tt-POP-1	2-Dichloroethane (DCE), formaldehyde dimethyl acetal (FDA), 1,4-bis(bromomethyl)benzene (BMB), and 4,4′-bis(bromomethyl)biphenyl (BBMP)	0.29	^168^
Tt-POP-2		0.49	
Tt-POP-3		0.45	
BILP-4		3.60	^169^
BILP-5		2.50	
BTLP-4		2.70	^170^
BTLP-5		1.98	
BOLP-4		2.00	
BOLP-5		1.80	
CQN-1g		4.57	^171^
CICF-KCl/NaCl-500		4.04	^172^
CICF-500		2.63	
Isox-CTF-5-400		2.86	^173^
HAT-CTF-450/600		4.80	^174^
Tz-df-CTF600		5.08	^175^
CTF-3		1.34	^176^
F_12_CTF-3		4.33	
O-CTF-3		1.70	
O-F_12_CTF-3		3.04	
O-CTF-2		2.06	
O-F_16_CTF-2		3.30	
F-DCBP-CTF-1		3.82	^177^
CTF-1		1.41	^178^
CTF-1-600		2.25	
FCTF-1		3.21	
FCTF-1-600		3.41	
NPOF-4		1.40	^179^
NPOF-4-NO_2_		1.56	
NPOF-4-NH_2_		1.88	
Azo-COP-1		1.48	^180^
Azo-COP-2		1.53	
Azo-COP-3		1.22	
Azo-COP-4		1.12	
Azo-COP-5		1.24	
Azo-COP-6		1.31	
Azo-COP-7		1.16	
Azo-COP-8		1.22	
Azo-COP-9		1.23	
Azo-COP-10		1.15	
Azo-COP-11		1.26	
Py-iPOP-1		0.55	^181^
cCTF-400		1.89	^182^
cCTF-450		1.41	
cCTF-500		1.82	
TMP1		2.04	^183^
TMP2		2.18	
TMP3		3.27	
TNHCP1		2.20	^184^
TNHCP2		2.11	
TNHCP3		2.23	
NTP		1.82	^185^
3D-CON		3.90	
CFX1-OH		1.50	^186^
NPOP		1.36	^187^
PNOP-1		2.42	^188^
PNOP-2		1.96	
3D-tPOP-NaCl-2.0		2.62	^189^
COP-210		2.85	^190^
COP-211		2.21	
COP-212		1.86	
COP-122		0.55	^191^
COP-122 ao		0.59	
SOF-7a		1.49	^192^
COP-115		1.02	^193^
COP-115-B		1.81	
COP-115-C		1.72	
COP-115-D		1.80	

#### 3.1.5 Membranes

Polymetric membranes that have physical characteristics of transparent glassy types or elastic rubbery types have been examined to capture CO_2_ under ambient conditions. Experimentation is tested under CO_2_/N_2_ selectivity which is the principle for DAC capture.^194^ Fujikawa has outlined a number of criteria simulated particularly for proficient DAC separation with a membrane. First, the feed stream which is the retentate is to be at 300 ppm of CO_2_ and 1 atm. Second, the permeate to vacuum is in the range of 0.01 bar. Lastly, multiple stages with a designed membrane are necessitated to achieve an acceptable CO_2_ concentration at the permeate.^195^ The focus of the late research is on the improvement of selectivity by functionalizing the membranes’ materials without losing permeability. This is achieved by following methods including engineering hybrid structures for the enhancement of physical durability, incorporating amine groups for the improvement of CO_2_ affinity, and optimizing the polymers–inorganic fillers interface in the mixed matrix membranes (MMMs).^196,197^ In addition to that, advanced fabrication methods like phase inversion and electrospinning are being explored for the purpose of performance enhancement.^198^ Anticipating the integration of catalytic systems with membrane separation for the conversion of CO_2_ can give a transformative method. This enables the simultaneous utilization of the captured CO_2_ to create value-added products such as fuels and chemicals, contributing to a higher level of sustainability in the carbon management cycle.^199^ The separation technology of membranes has the advantages of relatively low operational energy utilization and a small footprint. Nevertheless, the costs of the raw materials and manufacturing processes of membrane types differ drastically. MMMs or high-performance inorganic membranes imply larger early investments and higher maintenance costs. Conversely, polymer membranes are reasonably priced; however, their selectivity, fouling resistance, and lifespan impact the operational efficiency in the long run. For large-scale sources of CO_2_ emissions like steel mills or power plants, it is vital to take into account factors such as separation efficiency, membrane module replacement cycles, production line retrofitting costs, and operating energy consumption. The overall feasibility can be assessed appropriately by only satisfying particular criteria of separation. Membranes provide a comparatively continuous low-energy method for gas separation, and they are specifically attractive for CO_2_ capture in pre- and postcombustion processes.^200^ Table 5 shows a summary of membrane-based CO_2_ separation materials, and Table 6 shows a number of different occurrences of CO_2_ capture under ambient air by the membrane grouping.

**Table 5:** Membrane-Based CO_2_ Separation Materials

Membrane Type	Composition	Preparation Method	Permeability Selectivity	Advantages/Disadvantages	Application	Ref
Polymer (polyimide)	Pure polymer (e.g., cellulose acetate, poly(ether imide))	Phase inversion (solution casting/spinning)	Moderate CO_2_ permeability; moderate CO_2_/N_2_ selectivity	Low cost, easy processing	Mall-scale flue gas/natural gas purification	^201^
				Plasticizes at high pressure/temperature		
Inorganic (zeolite, ceramic)	Pure inorganic (e.g., zeolite, SiO_2_, Al_2_O_3_)	Chemical vapor deposition (CVD) or in situ crystallization	High selectivity; lower permeability than polymers	Thermally/chemically stable	High-temperature flue gas; specialty separations (H_2_/CO_2_)	^202^
				Brittle and expensive		
Mixed matrix membranes (MMMs)	Polymer matrix + inorganic fillers (MOF, zeolite, carbon)	Fillers dispersion in polymer solution	Tunable performance based on filler dispersion	Combines polymer flexibility with inorganic selectivity	Industrial-scale CO_2_ capture if dispersion issues resolved	^203,204^
				Interface compatibility challenges		
ZIF-8@PAN-PEO	PAN nanofibers loaded with ZIF-8; embedded in PEO	Electrospinning + solution blending	CO_2_ permeability increased by 52.4%; CO_2_/N_2_ selectivity up 44%	Mechanical strength	Postcombustion modular membrane separation	^205^
				Low mass transfer resistance		

**Table 6:** CO_2_ Adsorption Performance by the Assembly of Membrane^a^

Sorbent Material	Selectivity (CO_2_/N_2_)	CO_2_ Permeability	Ref
Polyimide PI-5	26	190 Barrer	^206^
P-polyetherimide	24.6	200 Barrer	^207^
PIM-1/Matrimid hollow fibers	27	217 GPU	^208^
UiO-66-NH2/PIM-1 MMMs	22	6000 Barrer	^209^
(PDAC/GO/DMAP-Au/PSS)_10_	48.5	24.84 GPU	^210^

a Permeability (*P*) in cubic centimeters (STP) centimeter/cubic centimeter seconds centimeter Hg × 10^–10^ (Barrer). 1 GPU = 1 × 10^–6^ cm^3^ (STP)/cm^2^ s cmHg = 7.5005 × 10^–12^ ms^–1^ Pa^–1^.

### 3.2 Chemical Sorbents

Chemical sorption is defined by the presence of a reaction forming chemical bonds between the sorbent and CO_2_ gas, resulting in a strong affinity of the sorbent to capture CO_2_. This makes chemically based sorbents more attractive materials for DAC applications. Amine-functionalized sorbents, solid alkali sorbents, and liquid chemisorbents are the main categories of this mechanism.^211^

#### 3.2.1 Amine-Functionalized Sorbents

Amine is widely used as a modification to solid adsorbents for DAC. Amines are well known for their reversible reactions with CO_2_, which make them idyllic for CO_2_ capture from a controlled stream of gas, depending on the requirement.^212^ Modification of solid adsorbents with amines involves three classifications. First is the physical impregnation of amine inside the volumetric structure of the porous basket. Second is creating grafting, which is a covalent linkage on the solid surface. Lastly, is *in situ* polymerization of amino polymers that are derived from amine-containing monomers onto the porous support.^213,214^ The analysis report has advocated that S-DAC is favored due to the lower energy required for regeneration. Solid sorbents have the capability to adsorb a high capacity for CO_2_ air capture at a low cost, with high selectivity and high stability. This is a good attribute for scale-up once the technology readiness level (TRL) has been accepted. Despite the moisture from the air, amine modification allows for capturing CO_2_ with no hindrance. However, amine-based solid adsorbents have high costs, limited existence due to time-dependent oxidative degradation, and lower kinetics.^19,51,55,215–217^ Table 7 reports some amine-modified sorbents and their CO_2_ sorption capacity.

**Table 7:** Amine-Modified Solid Sorbents for DAC of CO_2_ with the Adsorption Capacities^218^

Amine	Loading	Matrix	Adsorption Conditions	Capacity (mmol/g)
PEI	29.8/37.2/48.1 wt %	Mesoporous γ-alumina	400 ppm of CO_2_/Ar at 25 °C	1.03/1.33/1.74
PEI	24.8/39.9 wt %	SBA-15	400 ppm of CO_2_/Ar at 25 °C	0.32/1.05
PEI	45.1 wt %	Silica	400 ppm of CO_2_/Ar at 25 °C	2.36
A-PEI	46.0 wt %	Silica	400 ppm of CO_2_/Ar at 25 °C	2.26
T-PEI	45.0 wt %	Silica	400 ppm of CO_2_/Ar at 25 °C	2.19
PEI	5/10/30 wt %	Monolithic alumina	400 ppm of CO_2_/He at 30 °C	0.29/0.63/0.75
PEI	5/10/30 wt %	Powder alumina	400 ppm of CO_2_/He at 30 °C	0.34/0.57/0.71
PEI	30/40/50 wt %	SBA-15	400 ppm of CO_2_/He at 25 °C	0.65/1.23/0.57
PEI	27/40/47 wt %	Pore-expanded MCM-41	400 ppm of CO_2_/N_2_ at 25 °C	1.63/2.17/2.01
PEI	35/43 wt %	Calcined pore-expanded MCM-41	400 ppm of CO_2_/N_2_ at 25 °C	0.49/0.66
PEI	55 wt %	CA-SiO_2_ fiber	395 ppm of CO_2_/He at 35/45/55 °C	0.62/0.55/0.32
PEI	55 wt %	CA-SiO_2_ fiber	395 ppm of CO_2_/He at 35 °C/2–3 mol % H_2_O	1.71
PEI	0.51 g·g^–1^ silica	ePTFE/silica	400 ppm of CO_2_/He at 35 °C, 50% RH	1.5
PEI	50 wt %	Mg/Al mixed metal oxides	400 ppm of CO_2_/N_2_ at 25 °C	1.66
TEPA	67 wt %	Mg/Al mixed metal oxides	400 ppm of CO_2_/N_2_ at 25 °C	3.0
APS	0.69/1.52/2.91/4.13 mmol·g^–1^	Disordered alumina	400 ppm of CO_2_/N_2_ at 30 °C	0.18/0.18/0.32/0.61
APS	0.87/2.01/3.22/5.43 mmol·g^–1^	Ordered alumina	400 ppm of CO_2_/N_2_ at 30 °C	0.15/0.29/0.51/0.76
APS	3.75 mmol N·g^– 1^	Mesoporous silica foam	500 ppm of CO_2_/He at 25/45/65 °C	1.38/1.21/0.94
MAPS	2.41 mmol N·g^–1^	Mesoporous silica foam	500 ppm of CO_2_/He at 25/45/65 °C	0.4/0.2/0.09
PAA	30/50/70 wt %	Silica	450–470 ppm of CO_2_/N_2_ at 21 °C, 60–70% RH	0.287/0.716/4.27
EDA	Not reported	Porous polymer network	400 ppm of CO_2_/78.96% N_2_/21% O_2_ at 21.85 °C	0.15
DETA	Not reported	Porous polymer network	400 ppm of CO_2_/78.96% N_2_/21% O_2_ at 21.85 °C	1.04
DT	6/10/15	SBA-15	400 ppm of CO_2_/N_2_ at 45 °C	1.86/2.00/1.18
pH-3-EDA	27/37/43/51 wt %	SBA-15	400 ppm of CO_2_/He at 35 °C	0.42/0.96/1.43/0.9
pH-3-PD	34/43/53 wt %	SBA-15	400 ppm of CO_2_/He at 35 °C	0.25/0.72/1.23
pH-6-EDA	32/40/49 wt %	SBA-15	400 ppm of CO_2_/He at 35 °C	0.55/0.87/0.8
pH-6-PD	30/40/49 wt %	SBA-15	400 ppm of CO_2_/He at 35 °C	0.28/0.46/0.59
PL	2.76/4.84/5.18 mmol N·g^–1^	Brush–mesoporous silica	400 ppm of CO_2_/Ar at 25 °C	0.19/0.56/0.60
Aziridine	9.9/42.5 wt %	SBA-15	400 ppm of CO_2_/Ar at 25 °C, humid conditions	0.16/1.72
ED	5.5 wt %	Mg/DOBDC	400 ppm of CO_2_/Ar at 25 °C	1.55
mmen	Not reported	Mg_2_(dobpdc)	390 ppm of CO_2_/air at 25 °C	2.0
ED	N/Mg = 1.7	Mg_2_(dobpdc)	0.39 mbar CO_2_/air at 25 °C	2.83
TREN	5.67 mmol·g^–1^ MOF	MIL-101(Cr)	400 ppm of CO_2_/He at 24.85 °C	2.8
PEI	0.97/1.32/1.76 mmol·g^–1^ MOF	MIL-101(Cr)	400 ppm of CO_2_/He at 24.85 °C	1.15/1.2/1.35
PA	45.8 wt %	NbOFFIVE-1-Ni	400 ppm of CO_2_/N_2_ at 24.85 °C	1.44

#### 3.2.2 Mesoporous Silica

Mesoporous silica is an inorganic nanostructure that exhibits a honeycomb-like porous material. When this structure is modified, it can be functional depending on its purpose. There are different categories defining them: mesostructured silica nanoparticles (MSN), Mobil Composition of Matter-number (MCM-n), Santa Barbara Amorphous-number (SBA-n), and Korean Institute of Technology-6 (KIT-6).^38,219^ At a certain temperature and pressure, these structures are capable of providing a tunable large surface area and pore volume to facilitate CO_2_ capture.^220^ Table 8 shows a number of different occurrences of CO_2_ capture under ambient air by the mesoporous silica grouping.

**Table 8:** CO_2_ Adsorption Performance by the Assembly of Mesoporous Silica under the Conditions of 1 bar CO_2_ and 25 °C Temperature from Static Equilibrium Isotherms

Sorbent Material	Modification	Capacity (mmol/g)	Ref
Silica sphere	Aminopropyltriethoxysilane (APTES)	1.67	^221^
KIT-6 mesoporous Silica	Polyethyleneimine (PEI)	1.84	^222^
MCM-48 silica Xerogel	3-Aminopropyltriethoxy-silane	2.05	^223^
MCM-41	Propylethylenetriamine	0.97	^224^
Si-MCM-41	Polyethyleneimine (PEI)	0.62	^225^
Si-MCM-41-PEI-50		0.75	
TRI-PE-MCM-41	3-[2-(2-aminoethylamino) ethylamino]propyltrimethoxysilane (TRI)	2.65	^226^
Hierarchical bimodal meso/microporous silica	Polyethyleneimine (PEI)	3.36	^227^
Fumed silica	Polyethyleneimine (PEI) and polyethyleneglycol (PEG)	0.68	^228^
Silica	polymethylmethacrylate (PMMA) and tetra ethylene pentamine (TEPA)	2.50	^229^
TRI-PE-MCM-41	3-[2-(2-Aminoethylamino) ethylamino]propyltrimethoxysilane (TRI)	1.43	^230^
Hybrid silica material	3-Aminopropyl triethoxysilane and vinyl triethoxysilane	1.68	^231^
SBA-15 silica	Tetraethylenepentamine (TEPA)	2.30	^232^
SBA-15 silica	Polyethyleneimine (PEI)	1.34	
MIL-101 (Cr)	Tetraethylenepentamine (TEPA)	2.14	^233^
CARiACT G-10 silica	Tetraethylenepentamine (TEPA)	2.57	^234^
CARiACT G-10 silica	Polyethyleneimine (PEI)-600 (40 weight%)	1.53	
CARiACT G-10 silica	Polyethyleneimine (PEI)-600 (50 weight%)	2.36	
CARiACT G-10 silica	Polyethyleneimine (PEI)-1800 (50 weight%)	1.39	
PMC	Polyethyleneimine (PEI)-600 (50 weight%)	1.25	^235^
PME	Polyethyleneimine (PEI)-600 (50 weight%)	2.20	
Fumed silica	Polyethyleneimine (PEI)-600 (50 weight%)	2.44	^236^
Fumed silica	Polyethyleneimine (PEI)-1800 (50 weight%)	1.69	
PEI/Zr7-SBA-15	Polyethyleneimine (PEI)	0.85	^237^
SBA-15	N^1^-(3-trimethoxysilylpropyl)-Diethylenetriamine (DT)	0.13	^238^
W-AG-150A	N^1^-(3-trimethoxysilylpropyl)-Diethylenetriamine (DT)	1.97	
KIT-6	(3-aminopropyl) triethoxysilane (APTES)	1.56	^239^
SBA-15	Tetraethylenepentamine (TEPA)	3.23	^54^
SBA-15	Stepwise growth of amine-terminated melamine-based dendrimers	0.40	^240^
SBA-15	Aziridine	3.10	^241^
SBA-15	Polyethyleneimine (PEI)	0.18	
SBA-15	Uncalcined Tetraethylenepentamine	0.12	
SBA-15	Diamine	0.56	
SBA-15	NH_2_	0.55	
SBA-15	Poly(propylenimine) (PPI), HBr	0.25	^242^
SBA-15	Poly(propylenimine) (PPI), HClO_4_	0.31	
SBA-15	Poly(propylenimine) (PPI), HCl	0.15	
SBA-15	Poly(propylenimine) (PPI), CH_3_SO_3_H	0.18	
Amine-impregnated silica foam	Polyethyleneimine (PEI)	1.48	^243^

#### 3.2.3 Solid Alkali Sorbents

This class of sorbents is principally comprised of hydroxides, oxides, or carbonates of alkaline-earth and alkali metals, introducing a main material category for CO_2_ capture. This type normally operates within a chemisorption mechanism. Exploiting solid inorganic bases concept other than the absorbents (liquids) for the purpose of ultradilute CO_2_ capture was primarily established by Steinfeld and others.^244^ These materials were essentially designed for applications of high temperature like CO_2_ capture from flue gas in the postcombustion process. The current path is to adapt them for DAC of CO_2_. This growth is specifically connected to variations which allow regeneration at moderate level of temperature or utilize certain pathways of reactions. A significant benefit of this type of sorption materials is their high CO_2_ uptake theoretical values and their outstanding stability.^244^ In the presence of water, a reaction takes place between alkali metal oxides and CO_2_ forming carbonate or bicarbonate. For example, the chemisorption of CO_2_ by a potassium-based adsorbent (K_2_CO_3_) is a method with high selectivity for the diluted CO_2_ from the large amount of N_2_ in the air, according to the chemical reaction:^245,246^

$$K_{2}{\mathrm{CO}}_{3(\mathrm{solid})}+H_{2}O_{\left(\mathrm{gas}\right)}+{\mathrm{CO}}_{2(\mathrm{gas})}\to 2{\mathrm{KHCO}}_{3(\mathrm{solid})}$$

CaO-based sorbents are widely utilized for CO_2_ capture because of their commercial availability and low prices. Usually, CaO carbonation demands high temperatures for effective removal of CO_2_. Under ambient conditions, a prehydrated lime in DAC was investigated in order to improve the capture capability of CO_2_.^247^ The introduction of steam into the carbonation or calcination process demonstrated a positive influence on DAC performance. After CaO prehydration, the ratio of carbonation conversion was found to be higher than 50%. Faster carbonation of hydrated lime compared to lime is caused by two major phenomena: 1) more nonbound molecules of water in the hydrated lime enhance the dissolution of CO_2_ at the interface between the gas and solid, and 2) the formed CaCO_3_ on the CaO surface could be converted in the presence of water to Ca­(HCO_3_)_2_. This compound breaks the CaCO_3_ layer protection which improves the diffusion of CO_2_ through the particle bulk. Also, sodium and potassium (Na, K)-based alkali metal carbonates/oxides can be utilized for capturing CO_2_. CO_2_ partial pressure, temperature, and H_2_O partial pressure affect the chemical reaction between CO_2_ and alkali metals. Alkali carbonates loaded into porous materials are investigated to overcome the low carbonation rates problem when the concentration of CO_2_ in the air is ultradiluted. K_2_CO_3_ compound was extensively introduced to various porous matrices. In the CO_2_ presence, K_2_CO_3_ is converted further into KHCO_3_ to capture CO_2_. Within the moist environment, K_2_CO_3_ compound experiences hydration reaction in which the unstable compound potassium carbonate sesquihydrate (K_2_CO_3_·1.5H_2_O) is forme, and the final compound KHCO_3_ is produced rapidly.^218^ Kim et al. studied the CO_2_ capacity performance of solid sorbents based on alkali and alkaline earth metal oxides. The study was performed under ambient conditions. They showed that Na_2_O exhibits superior desorption ability avoiding the need for steam generation . Table 9 shows the results of different materials and conditions for adsorption–desorption cycles using alkaline sorbents combined with 10% oxides of alkali and alkaline earth metals.^248^ Also, Table 10 shows a comparison of various alkali metal sorbents in DAC applications . Moreover, Table 11 compares the CO_2_ adsorption–desorption performance of different potassium-based adsorbents.

**Table 9:** Moist Adsorption and Dry Desorption Examination in a Temperature Swing Process Using Alkaline Sorbents Each Containing 0.25% Ru Combined with 10% Oxides of Alkaline Earth Metals and Alkali (Na_2_O, K_2_O, MgO, BaO, and CaO) Supported on Al_2_O_3_ ^a^

		CO_2_ Capacity (μmol/g)				
Desorption T/°C	Conditions	Na_2_O	K_2_O	MgO	CaO	BaO
RT	All samples were pretreated by catalytic hydrogenation	420	150	95	55	65
100		100	20	35	20	25
125	Adsorption: 400 ppm of CO_2_/air + 2 mol % H_2_O	40	10	5	2	3
150		60	5	2	1	2
175	Dry desorption in N_2_ was conducted from ambient to 200 °C at 5 °C/min heating rate	105	15	3	2	2
200		190	30	5	2	2
RT	Adsorption: 400 ppm of CO_2_/air + 2 mol % H_2_O	570	460	200	95	90
100		165	90	70	40	50
125	Moist desorption in N_2_ was conducted from ambient to 200 °C at 5 °C/min heating rate with 2 mol % H_2_O.	130	50	15	5	10
150		95	60	15	5	3
175		95	100	15	5	3
200		70	160	20	5	3

a Adsorption stage was conducted at room temperature (RT), while desorption at a range of RT to 200 °C.^248^

**Table 10:** Various Alkali Metal Sorbents in DAC Applications

Alkali metal	Support	CO_2_ concentration	Adsorption capacity (mmol/g)	Reference
K_2_CO_3_	Carbon	410 ppm	0.70	^249^
Ni-CaO	γ-Al_2_O_3_	5% CO_2_/N_2_	0.07	^250^
Ni-Na_2_O	γ-Al_2_O_3_	5% CO_2_/N_2_	0.2	
Ru-CaO	γ-Al_2_O_3_	400 ppm	0.47	^251^
Na_2_O	γ-Al_2_O_3_	400 ppm	0.4	^252^
CaO	HcATP	400 ppm	0.67	^253^

**Table 11:** CO_2_ Adsorption–Desorption Performance Comparison of Different Potassium-Based Sorbents

Support	K_2_CO_3_ (wt %)	Capacity (mg/g)	Adsorption Conditions	Desorption Conditions	Cycle	refs
MgO@TiO_2_	50	126.6	450–500 ppm of CO_2_, 50 RH %, 22–25 °C	300 °C	20	^245^
MgO	30	120	1 vol % CO_2_, 9 vol % H_2_O, 60 °C	350–400 °C	5	^254^
γ-Al_2_O_3_	21–23	27	Cooling step for 6 h with ambient air	300 °C	25	^255^
Al_2_O_3_	24.5	74	13% CO_2_, 13% H_2_O, 60 °C	350 °C	10	^256^
Al_2_O_3_	36.8	93	10% CO_2_, 14% H_2_O, 60 °C	300 °C	80	^257^
Al_2_O_3_	36.8	112	3–20 vol % CO_2_, 8–16 vol % H_2_O, 60 °C	250 °C	20 h operation	^258^
TiO_2_	35	83	8–11.2 vol % CO_2_, 7–30 vol % H_2_O, 60–90 °C	120–220 °C	20 h operation	^259^
TiO_2_	30	83–92	1 vol % CO_2_, 9 vol % H_2_O, 60 °C	150 °C	10	^260^
ZrO_2_	30	91.6	1 vol % CO_2_, 9 vol % H_2_O, 60 °C	150 °C	10	
Activated carbon	33.3	92	1 vol % CO_2_, 9 vol % H_2_O, 60 °C	200 °C	10	^261^
Activated carbon	24	73.4	15% H_2_O, 15% CO_2_, 60 °C	200 °C	1	^262^
Hydrotalcite	62	78.7	1 bar pure CO_2_, 300 °C	500 °C	8	^263^

#### 3.2.4 Liquid Chemical Absorbents

The technology of amine scrubbing is the utmost matured technology to capture CO_2_ gas from flue gas and natural gas.^264^ The most widely utilized aqueous amine solutions in the CO_2_ absorption industry are monoethanolamine (MEA), diethanolamine (DEA), and methyldiethanolamine (MDEA).^265^ Amine-based solutions showed the advantages of high reaction rates and reasonable absorption capacity for CO_2_ gas, in addition to their low costs. However, their major disadvantages are the high heat requirements for regeneration and the formation of hydroperoxides which is known as oxidative degradation.^266,267^ The high energy consumption during amine solution regeneration majorly originates from the loss of sensible heat of water. Hence, water-free or water-lean chemical absorption gained a lot of attention.^268–270^ The potential amine degradation causes equipment corrosion, solvent loss, and the generation of volatile degradation compounds. These issues may be harmful to the environment and human beings’ health.^271^ The efficient absorption of CO_2_ from streams containing ultradilute ambient air requires proper materials with enough level of stability and low energy intensity.^272^ The most suitable materials reported as effective aqueous alkali hydroxide sorbents for DAC systems were the aqueous unhindered primary amines.^273^ These materials are promising for high-efficiency energy savings due to their lower sorbent regeneration temperature. Blends of amines have the ability to combine the advantages of each component which may give better results for DAC systems.^265^ Additionally, ionic liquid materials and phase-changing liquids were explored for DAC applications.^218^

### 3.3 New Material Directions for DAC Applications

The research will progressively focus on realistic design principles to manufacture a new generation of sorption materials with extraordinary efficiency. This includes the development of hybrid and multifunctional materials combining several functionalities like dual-function materials which capture and convert CO_2_ within space missions for life support,^274^ or integrated CO_2_ capture and utilization (ICCU).^34,275,276^ Hybrid materials, such as polymer-sorbent composites^277,278^ or MOF-ionic liquid composites,^279^ are able to influence the strong points of several components. Copying inspiration from the natural processes of CO_2_ capture and the utilization of biomass-derived materials in the production of sustainable and bioinspired sorption materials^280^ results in more cost-effective and sustainable sorbents. Moreover, smart and responsive sorbent designs that are able to regulate their CO_2_ selectivity or affinity in response to external stimuli such as humidity, temperature, and electrical fields can facilitate more flexible and effective DAC processes,^281^ including the exploration of synergetic sorption mechanisms.^282^ Additionally, tailored surface chemistry and porosity at the nanolevel achieved through accurate control of surface area, pore size distribution, and chemical functionalization will be pivotal for the CO_2_ diffusion optimization, water tolerance, and binding strength, including the enhancement of amine grafting methods and superhydrophobic materials.^244^

To synthesize the extensive performance metrics detailed across the diverse sorbent classifications, Figure 3 provides a comparative benchmarking of the highest-performing materials within each solid sorbent category. This visualization illustrates the exceptional equilibrium CO_2_ uptake capacities achieved by chemically activated carbon-based materials and metal-exchanged zeolites, contrasting them against the upper-bound capacities of advanced metal–organic frameworks and mesoporous silicas. Ultimately, these benchmarks underscore the critical thermodynamic advantage of integrating targeted basic functionalities such as amine tethering or alkali metal doping into highly tunable porous scaffolds to overcome the ultradilute partial pressures of atmospheric CO_2_. Table 12 shows an overall comparison between the types of sorbents.

**Figure 3:**
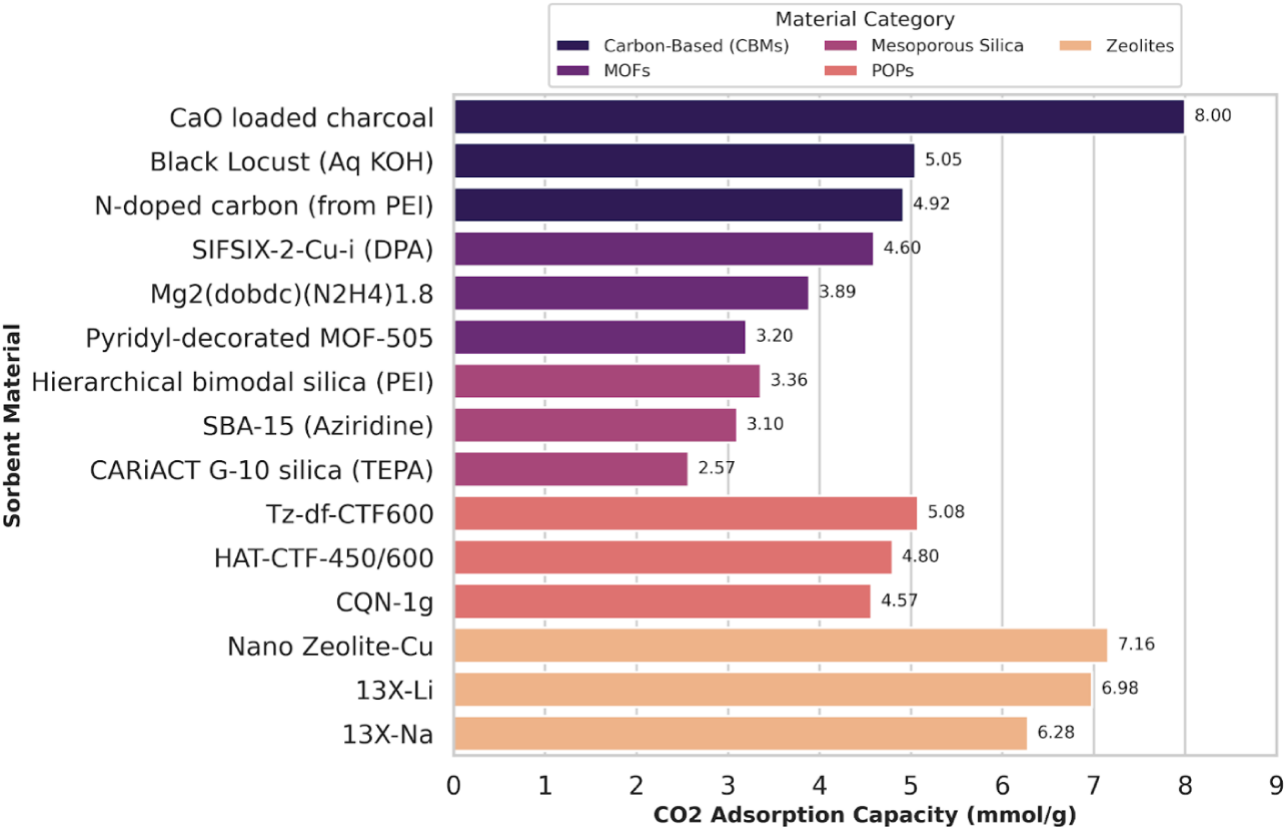
Comparative equilibrium CO_2_ adsorption capacities (mmol/g) of the top-performing solid sorbents under ambient DAC conditions. Data highlights the top three materials identified within mesoporous silicas, zeolites, CBMs, MOFs, and POPs. Membrane separators are excluded from this specific comparison, as their operational efficiency is dictated by kinetic permeance and selectivity rather than absolute thermodynamic equilibrium capacity. The adsorption conditions are 1 bar CO_2_ and 25 °C temperature from static equilibrium isotherms.

**Table 12:** Comparison of Different Sorbent Classes for DAC^218^

Specifications	Physical Sorbents	Chemical Adsorbents	Chemical Absorbents
Capacity	Low capacity for most physical adsorbent	High	High
	High capacity for certain MOFs		
Sorptionkinetics	Relatively low kinetics Competitive for some MOFs	Moderate	Fast
Performance under moisture conditions	Reduced capacity for the most physical adsorbents	Improved capacity in presence of less water	Improved capacity in presence of less water for water-lean or water-free absorbent, e.g., ionic liquid
	Improved capacity in presence of less water for several MOFs		
	Unstable for MOFs		
Regeneration energy	Relatively low	Moderate	Significant
Advantages	Mature, cheap, stable, and easily available for zeolite and AC	Great potential to be modified	High reaction rate
	High modification flexibility for MOFs	Lower energy consumption than aqueous solution	Reasonable CO_2_ capacity
			Low cost
Disadvantages	Poor capacity and selectivity	High cost	High regeneration cost
	High cost, instability and toxic raw materials for MOFs	Unstable after thousands of cycles	Oxidative degradation
	Poor performance under moisture conditions		Solvent loss
			Corrosion

## 4 Process Engineering in DAC of CO_2_

Recovery of the material used to obtain the original functionality to retain the performance value is directly proportional to the economic implementation of the whole procedure. Few approaches can be contemplated, and these will be discussed further in the subsequent subsections.

### 4.1 Pressure/Vacuum Swing Processes

Pressure swing adsorption (PSA) implicates a method to pressurize the contained cell area after the completion of the adsorption experiment. This usually requires a particular selected gas in the inlet to be compressed under a certain pressure to purge the CO_2_ trapped in the raw materials. Once the system has reached equilibrium by perceiving the pressure drop within a certain time limit, it indicates that the CO_2_ has eluded from the sorbates. Vacuum swing adsorption (VSA) comprises a certain vacuum installed on the exit side to withdraw all the trapped CO_2_ after the adsorption experiment. This method is more stable, as it does not involve any sudden rush of compressed gas as opposed to the PSA system. Recovery is also dependent on the duration of the VSA operation, signifying that a longer time relates to more efficiency in extracting the CO_2_ for regeneration. Pressure-vacuum swing adsorption (PVSA), which is a combination of both stages, involves an adsorption process conducted at a pressure higher than atmospheric pressure, followed by a vacuum operation performed at a pressure lower than atmospheric pressure. Likewise with PSA, this PVSA method is also not favorable due to the compression of gas in a contained area.^35,283–285^ VSA seems to be a more practical option in this section; however, it can also be challenging, as prolonged vacuuming operations incur additional costs. Finally, if time is of the essence in the overall operation, this would be a debatable option.

### 4.2 Temperature Swing Processes

Treasure swing adsorption (TSA) implicates setting a certain high temperature in the cell after the chemisorption process. This allows the breaking of chemical bonds in the trapped CO_2_ within the sorbates, exiting the premises of the cell. Three ways can be well though-out for this operation: as-is cell, inert gas inlet, pure CO_2_ gas inlet, and saturated steam. As-is cell gas during the TSA process means that no particular gas is introduced into the inlet during the operation. When the temperature rises, CO_2_ molecules will be released from the sorbates that can cause oxidative degradation at certain high temperatures; hence, this option is not advisable. Inert gas is somewhat better compared to an empty cell during TSA operation; however, the downfall lies in its cost. Pure CO_2_ is also one of the options, which is good in terms of cost, yet at certain raised temperatures, it can cause urea formation that can be damaging to the inner cell. Saturated steam is considered as well at the inlet; however, this can strip the sorbates to an uncomplementary state that it does not function at optimum anymore.^35,286–292^ Temperature-vacuum swing adsorption (TVSA) is more feasible for regeneration. Similar to TSA, where the temperature is being raised to distort the chemical bond between the CO_2_ molecule and the sorbates, parallelly the released CO_2_ is being forced out of the system via vacuum. As good as it sounds, the raised temperature in a vacuum cell can lead to the thermal degradation of the sorbents, limiting their further usage. This setback nevertheless can be advocated by the presence of an inert gas in the inlet section during the TVSA operation. This suggests that with an inert gas entering through the inlet, while the temperature is raised in the cell and vacuuming occurs simultaneously, the regeneration process becomes more satisfactory.^35,288,293–295^ Figure 4 provides a comparison between TSA and TVSA for CO_2_ capture.

**Figure 4:**
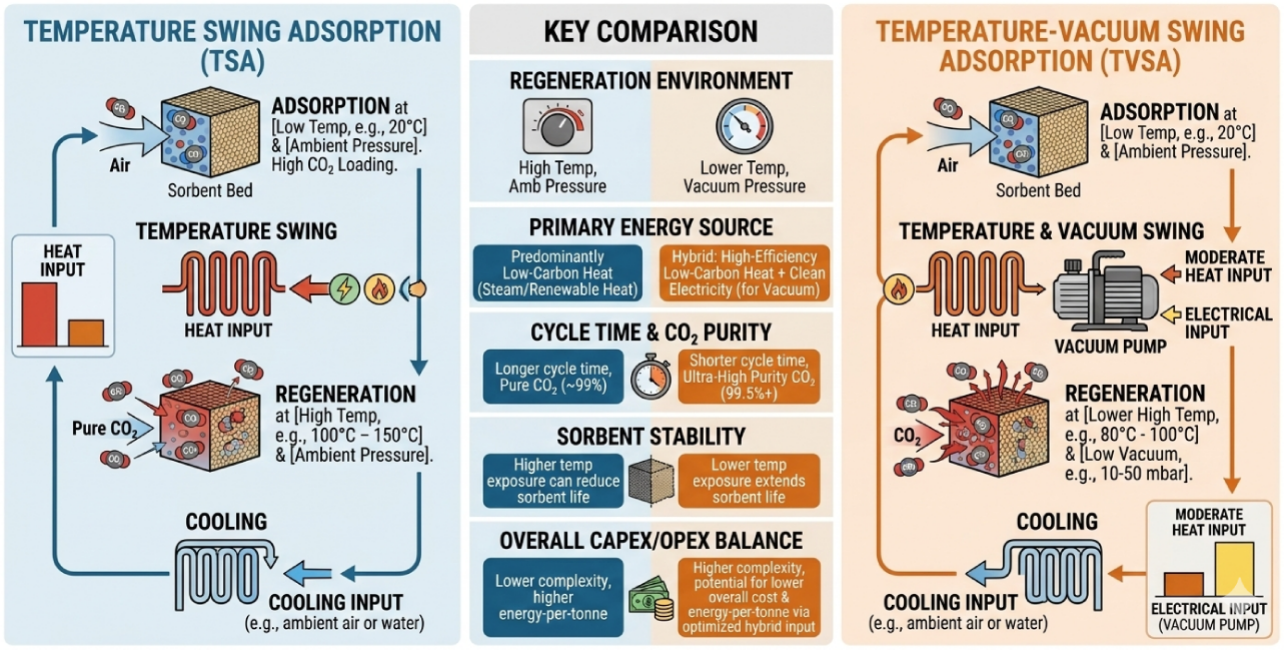
A comparison of TSA and TVSA CO_2_ capture cycles, highlighting the differences in energy inputs, cycle time, CO_2_ purity, and overall system intricacy.^35,229,235,283–286,288–291,293,294^

### 4.3 Calcium-Based Kraft Process

This method of regeneration falls under the L-DAC sorbent, which involves an absorption process. The idea of using an alkaline system impersonating the Kraft process to capture CO_2_ was adapted by Carbon Engineering. The capturing process starts with a mixture of an alkaline-based solution: sodium hydroxide (NaOH) or potassium hydroxide (KOH) and calcium hydroxide (Ca­(OH)_2_) reacting with trapped CO_2_ from ambient air to form calcium carbonate (CaCO_3_). All of the steps are exothermic, which is favorable to capture CO_2_ from air until the final product CaCO_3_. However, in the stage of the recovery process, withdrawing CO_2_ from CaCO_3_ is an endothermic process. This leads to involving more energy to regenerate the Ca­(OH)_2_ in the capturing stage with additional calcium oxide (CaO) and water (H_2_O) in the recovery stage. Carbon Engineering has observed this and opted for KOH as their alkaline-based solution to increase the competence of the whole process.^35,286,296^

### 4.4 Moisture Swing Adsorption

This method of regeneration involves fine-tuning the relative humidity in the process of CO_2_ capture and release. This concept proposes the capture of CO_2_ under dry conditions. This is then followed by releasing it under humid conditions, using materials like anion exchange resins, often with quaternary ammonium groups, which absorb water to trigger CO_2_ desorption. The idea of no heat usage is relatively acknowledging that this is a low-cost approach. Its utilization as the recovery of CO_2_ with relative humidity can be determined depending on their requirements. The challenge here lies only in the condition of locking in the CO_2_ during the capturing process where it has been really dry.^35,297–299^ Tables 13 and 14 cover the main parameters of solid and liquid sorbents under full DAC condition cycles based on different regeneration methods. The energy values summarized in Tables 13 and 14 provide a practical, process-relevant perspective on the effective strength of CO_2_ binding in DAC systems, particularly at industrially relevant operating conditions. While adsorption enthalpy and isosteric heat offer valuable thermodynamic insight at the material level, such quantities are not uniformly reported across DAC studies and are often difficult to define for chemisorption-based solids and liquid sorbents due to multiple binding modes, moisture effects, and cooperative reaction pathways. In contrast, regeneration energy directly reflects the net energy penalty required to release CO_2_ and restore sorbent capacity under cyclic DAC operation. As shown in Tables 13 and 14, the reported thermal and electrical energy requirements for both solid and liquid systems fall within ranges characteristic of chemisorption-dominated capture, consistent with the thermodynamic considerations discussed in Section 2. These data therefore serve as a complementary and experimentally accessible framework for evaluating the sorbent performance and feasibility in practical DAC applications.

**Table 13:** Main Parameters under Full DAC Condition Cycles of Solid Sorbents Based on Different Regeneration Methods

Sorbent Material	Regeneration Method	Desorption Capacity (mmol/g)	Adsorption Conditions	Desorption Conditions	Energy (kJ/mol)	Stability	Ref.
γ-Alumina-PEI	TCS with steam and N_2_ purging	1.7	400 ppm, 30 °C and 50% RH	115 °C	-	Capacity was reduced to 0.66 mmol/g after 24 h because of amine leaching	^289^
Mesoporous carbons-PEI	TCS-N_2_ purging	2.58	400 ppm, 25 °C and 80% RH	110 °C	-	3% drop in adsorption capacity after 10 cycles	^300^
Fumed silica-PEI-33	TVSA	1.74	420 ppm, 25 °C and 67% RH	85 °C, 0.086 mbar	-	3.5% capacity reduction after 4 cycles. No degradation due to the oxygen	^301^
SI-AEATPMS	TVS	0.2–0.26	400–440 ppm, 25 °C, 40% RH	74–90 °C, 50–150 mbar	430 thermal, 9.6 mechanical	Stable capacity of 0.19–0.17 mmol/g over 40 consecutive adsorption/desorption cycles	^302^
SI-AEATPMS	TCS	0.45	400–440 ppm, 25 °C, 40% RH	90 °C	430 thermal	No degradation due to the urea groups occur, as other TCS cycles	
AEAPDMS-NFC-FD	TCS with argon	1.39	506 ppm, 25 °C, 40% RH	90 °C	-	Over 20 consecutive 2-h adsorption/1-h-desorption cycles, yielding a cyclic capacity of 0.695 mmol CO_2_/g	^303^
APDES-NFC	TVS	2.13	400 ppm, 25 °C and 80% RH		97.6–390.4 thermal	Cyclic capacity of 1 mmol/g over 100 TVS cycles	^304^
Mg0.55Al-O-TEPA67%	TCS with N_2_	3	400 ppm, 25 °C	100 °C	-	They simulate cyclic stability of 2.31 mmol/g using TSA	^305^

**Table 14:** Main Parameters under Full DAC Condition Cycles of Liquid Sorbents Based on Different Regeneration Methods

Sorbent Material	Regeneration Method	Capacity	Adsorption Conditions	Desorption Conditions	Energy (kJ/mol)	Ref
NaOH-Ca(OH)_2_ option A	Kraft process	0.42 MtCO_2_/y	500 ppm, ambient	900 °C	387 thermal and 79.2 electrical	^306^
NaOH-Ca(OH)_2_ option B	Kraft process	0.42 MtCO_2_/y	500 ppm, ambient	900 °C	264 thermal and 70.4 electrical	
NaOH-Ca(OH)_2_	Kraft process	-	380 ppm, ambient	900 °C	319 thermal and 123 electrical	^307^
NaOH-Ca(OH)_2_	Kraft process	-	400 ppm, ambient	900 °C	190–390 thermal	^308^
KOH-Ca(OH)_2_	Kraft process	0.98 Mt-CO_2_/year	400 ppm, 20C and 64% RH	900 °C	178.2 thermal and 63.7 electrical	^309^
NaOH-Na_2_O_3_·TiO_2_	-	-	400 ppm	860 °C	150 thermal	^310^
NaOH-H_2_-recycling electrochemical	Electrochemical	-	DAC conditions	-	374 thermal	^311^
Glycine amino acids-PyBIG	Crystallization	0.28 mol/mol cyclic capacity	Ambient conditions	8–120 °C	223 thermal and without water 75 thermal	^312^
H_2_O-Trichelating iminoguanidine ligand (BTIG)	Crystallization	0.99 mol/mol CO_2_ uptake	Ambient conditions	60–150 °C	169 thermal and without water 81thermal	^313^
Liquid AAS hydrogel particles (LAHPs)	-	0.96 mmol/g CO_2_ uptake	420 ppm of CO_2_	40–90 °C	-	^314^

## 5 DAC System Design and Contactor Technologies

Air contactors involve huge, engineered structures that were precisely constructed to draw ambient air and pass it through a selected material. This is followed by the release of CO_2_ and allocates the depleted concentration of CO_2_ back into the air. This acts similarly to how a tree or plant works; however, the footprint of utilizing the land is much larger for DAC.^315^ Figure 5 shows different air contactors.

**Figure 5:**
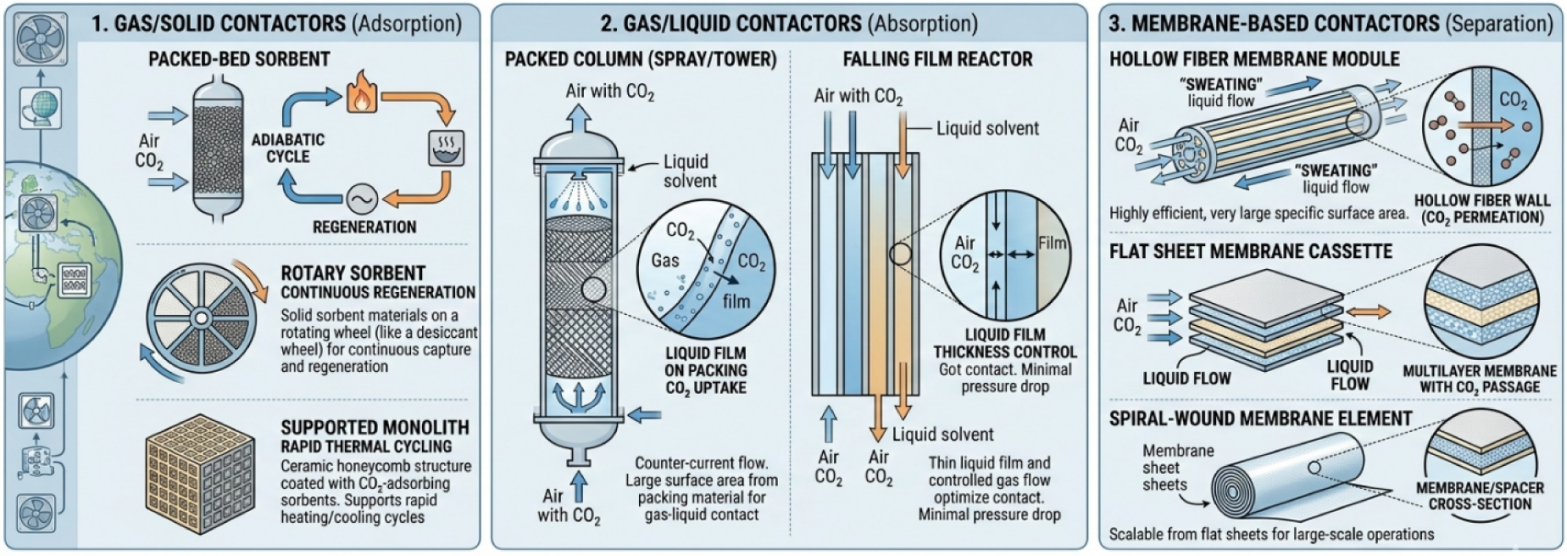
Air contactors for CO_2_ capture: gas–solid, gas–liquid, and membrane-based systems.^19,29,194,195,309,309–322^

### 5.1 Gas–Solid Contactors

Air-to-solid constructions customarily imply air flowing to a stationary bed of solid sorbents. The operation starts by allowing ambient air to be drawn into a designated space where the solid material is awaiting to start adsorbing CO_2_, thereby separating it from the air. Once equilibrium is attained, usually quantified by pressure and temperature conditions, the desorption process starts taking place. The contractor stops the process and allows the designated area to be vacuumed. The vacuuming process is crucial to be undergone to avoid degradation of the amine-modified sorbents. This can be explained by the fact that lingering oxygen and nitrogen are capable of contributing to the degradation of the amine-modified sorbents. Regeneration of the material which falls within the range between 80 and 120 °C takes place in the next sequence, allowing the removal of trapped CO_2_ from the material itself as well as from the contractor area prepared to be utilized again.^19,316,317^

### 5.2 Gas–Liquid Contactors

This type of mechanism involves air being drawn by the reactor that mixes the CO_2_ from the ambient air to allow contact with the liquid sorbents. For the gas–liquid mechanism, five key factors are taken into thoughtful execution for smooth operation: 1) contact angle for wettability measurement, 2) high surface area, 3) pressure drop, 4) cost related to raw materials and construction, and 5) maintenance. Contact angle is a type of measurement that computes the surface or the material wettability. Contact angles are preferred to be on the low side, allowing the solvent to distribute impeccably while adhering to the surface with good absorption.^318–320^ CO_2_ removal per unit volume is much more efficient with a high surface area-to-volume ratio for the construction inside the space where the ambient air is in contact with the solvent. Pressure drop plays a key role inside the system when the gas stream and the liquid solvent enter. The pressure difference between these two flows is ideally kept on the low side to avoid extra energy for pumping that ultimately means higher costs.^309,316,321^ Solvent-based air capture requires proper corrosion-resistant infrastructure and the stability of the raw materials. Hence, these categories must be evaluated to achieve an economically practicable operation and avoid high costs.^322^

### 5.3 Membrane Contactors

Membrane separators for CO_2_ removal are considered in the DAC field due to their lower footprint and minimal effort in operation. For membrane-DAC (m-DAC) operation, factors such as 1) permeance, 2) selectivity, 3) pressure ratio, and 4) stage cut (Ø) are crucial. Permeance, a process which allows selective solutes in a stream to penetrate through a membrane is the first factor considered. High-permeance separation membranes can be considered as a practical option for DAC since the membrane area is inversely proportional to the gas permeance value. Selectivity is also one of the key factors since the partial pressure of CO_2_ is much lower compared to flue gas. This consequently leads to a decision regarding how many stages with a “distinct membrane and its meticulous area” are required to achieve an effective DAC process. When the stages have been developed theoretically, the pressure ratio is calculated at each stage between the retentate gas and permeate gas. With this computation, stage cut which is defined by the ratio of permeate to retentate flow allows us to gain control in tuning the membrane area and the flow rate. All these factors are important for a transaction of the purity and the recovery of CO_2_ through the m-DAC process.^194,195^

## 6 Industrial-Scale DAC Technologies

To contextualize the rapid commercialization of direct air capture (DAC) technologies, Figure 6 illustrates the global landscape of operational companies and pioneering scale-up facilities. The United States currently hosts the highest concentration of active DAC enterprises, followed by significant innovation hubs across Europe and Canada. This combined geographical and quantitative visualization highlights the regional concentrations of technological development. Furthermore, the map denotes the physical deployment of flagship commercial plants that are currently driving industrial scale-up. The specific operational mechanisms, regeneration strategies, and geographic milestones of these industry leaders, including Climeworks, Carbon Engineering, and Global Thermostat, are detailed in the following subsections.

**Figure 6:**
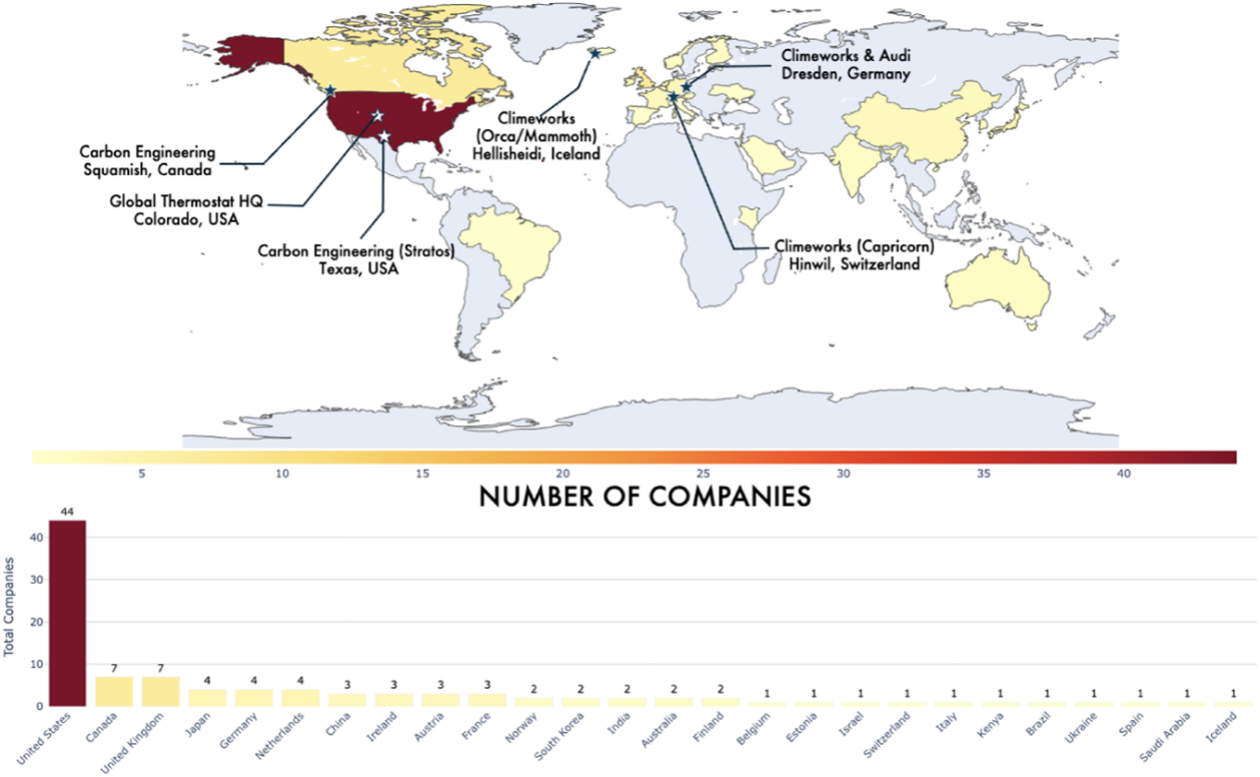
Global distribution of operational DAC companies and major industrial scale-up sites. The choropleth map (top) and corresponding quantitative bar chart (bottom) illustrate the concentration of active DAC enterprises by country.

### 6.1 Climeworks, Switzerland

Two freshmen in the field of engineering, Christoph Gebald and Jan Wurzbacher, passionately active in alpine sports, observed the glaciers declining. Their discerns drove them to counter this setback and led to the creation of the first operational DAC company, Climeworks. It is now one of the global leaders in DAC technology with a holistic approach. Their motto, “Climeworks lives and breathes DAC” drives them toward excellent engineering designs that are scalable, efficient, and measurable.^323^ They operate on a low-temperature swing process and utilize cellulose filter fibers equipped with amine-customized solid sorbents. These solid sorbents trap moisture from the air while also generating water. The whole operation takes 4–6 h with cost-conscious energy usage and 99.9% CO_2_ removal.^19,323,324^ From the beginning in 2009, when the concept was introduced and investors were secured for the development of a prototype, the first operational prototype at the pilot plant scale was tested in 2014. In 2017, Climeworks commissioned its first DAC commercial plant named “Capricorn” in Hinwil, Switzerland. This was a revolution, being the first plant to evolve from lab-scale to real-world application. It was followed by two plants, “Arctic Fox” and “Orca”, where each operation involves CO_2_ capture and underground storage. “Mammoth”, the latest and largest DAC plant to date, also operates with CO_2_ capture and underground storage.^21,323^ Climework associates with Audi to develop CO_2_-neutral synthetic fuels by capturing 80% of CO_2_ molecules from the air passing through the DAC system and the plant is located in Dresden, Germany. Climeworks is targeting production costs of about 75 €/t CO_2_ for large-scale plants in the long run to assist in achieving net zero 2050.^19,325^

### 6.2 Carbon Engineering, Canada

David Keith and Geoff Holmes pioneered an affordable DAC by adapting existing chemical processes for industrial scale. This company operates on the basis of high-temperature swing operation and utilizes solvents to absorb ambient air. In addition to that, it operates on a closed-loop solvent system in a continuous operation leading to an economical approach of reusing the solvent solution. Influential investors join in to have an inclusive goal of establishing broad commercial deployment of synthetic fuels production based on DAC technology. This is defined as Air to Fuel (A2F), and the first synthetic fuel plant was launched in 201. Carbon Engineering partners with oxy/1PointFive commence the “STRATOS” project in the Permian Basin, Texas, being the first commercial scale project of the company. Carbon Engineering intends to achieve costs of 75–113 €/t CO_2_ captured, purified, and compressed to 150 bar.^19,326,327^

### 6.3 Global Thermostat, USA

Global Thermostat, founded by Eisenberger in 2010, has been operating since then under the category of temperature swing solid sorbents. The company has multifunctional technology capable of capturing CO_2_ from both the atmosphere as well as point source emissions. In 2024, it merges with ZeroCarbonSystem to achieve highly scalable, low-cost, and low-energy carbon dioxide removal at a megaton-plus scale. The company has revealed its priorities and promises to deliver CO_2_ at a cost of 11–38 €/t CO_2_ and remove 500 million tons of CO_2_ per year from the atmosphere by 2050 as part of the net-zero plan.^19,328^

### 6.4 Emerging DAC Systems

Multiple systematic financial investments are ongoing in different DAC technologies around the world. Table 15 below catalogs diverse DAC companies around the globe based on location and sorbents classification, which subsequently mentions the method of operation and their regeneration.^329^

**Table 15:** List of Operational DAC Companies to Date^329^

Company Name	Location	Sorbent Type	Regeneration
280 Earth	USA	Solid	Temperature and vacuum
8 Rivers	USA	Solid	Temperature
ADNOC	Unspecified	Unspecified	Unspecified
Advanced Cooling Technologies	USA	Solid	Temperature or electrochemical
Advanced Energy Materials	USA	Solid	Plasma
Aeon Blue	Canada	Liquid	Electrochemical
Aerleum	France	Solid	Temperature
Aetherworks	USA	Not specified	Not specified
Air to Earth	USA	Solid	Temperature and vacuum
Air View Engineering	United Kingdom	Solid	Temperature and vacuum
Airbus	The Netherlands	Solid	Temperature
Aircapture	USA	Solid	Temperature and vacuum
Aircela	USA	Liquid	Electrochemical
Airhive	United Kingdom	Solid	Temperature
AirMyne	USA	Liquid	Temperature
Alitic	USA	Liquid	Chemical
Aramco	Saudi Arabia	Not specified	Not specified
Arbon	USA	Solid	Moisture
AspiraDAC	Austria	Solid	Temperature and vacuum
Atmosfuture	United Kingdom	None	Temperature (cryogenic)
Atoco	USA	Solid	Temperature
Avnos	USA	Solid	Moisture and vacuum
BluSky Carbon	Canada	Not specified	Not specified
C-Fix	USA	Solid	Electrochemical
Capture6	USA	Liquid	
Capture Tower	Spain	Not specified	Temperature
Carbominer	Ukraine	Solid	Electrochemical
Carbon 1.5	France	Solid	Not specified
Carbon Blade	USA	Liquid	Electrochemical
Carbon Capture and Commercialization	USA	Solid	Temperature
Carbon Collect	Ireland	Solid	Temperature and vacuum or moisture
Carbon Energy	South Korea	Not specified	Not specified
Carbon Engineering	USA	Liquid	Temperature
Carbon Reform	USA	Solid	Not specified
Carbon Utility	USA	Liquid	Electrochemical
CarbonAir Energy	Brazil	Solid	Not specified
CarbonCapture Inc.	USA	Solid	Temperature and vacuum
Carbon Corp	USA	Liquid	Electrochemical
Carbon To Stone	USA	Liquid	Chemical
Carbon Xtract	Japan	Membrane	Temperature or electrochemical
Carbyon	The Netherlands	Membrane	Temperature
CarpeCarbon	Italy	Not specified	Not specified
China Energy Engineering Corporation	China	Not specified	Not specified
Clarity Technology	USA	Solid	Temperature
Clean Capture Tech	Unites States	Solid	Temperature
Climeworks	Switzerland	Solid	Temperature and vacuum
Climatech Environment	India	Liquid	Not specified
CO_2_ CirculAir	The Netherlands	Membrane	Electrochemical
CtrlZ Climate	USA	Not specified	Not specified
DAC City	USA	Solid	Moisture and temperature
DACLab	USA	Solid	Temperature
DACMA GmbH	Germany	Solid	Temperature and vacuum
Decarbon	USA	Solid	Temperature
DeCarbon Tech	China	Solid	Not specified
Direct Carbon	Sweden	Not specified	Not specified
EDAC Laboratories	USA	Liquid	Chemical
Equatic	USA	Liquid	Not specified
E-quester	Canada	Liquid	Electrochemical
Equinor	United Kingdom	Liquid	Not specified
ExxonMobil	USA	Solid	Not specified
Feather Fuels	USA	Not specified	Not specified
Flow Aluminum	USA	Liquid	Electrochemical
Freshean	USA	Solid	Not specified
Fugu	Australia	Solid	Temperature
Gaia Refinery	Canada	Liquid	Chemical
GE Vernova	USA	Solid	Temperature and vacuum
GigaDAC/Victory Over Carbon	USA	Liquid	Not specified
Giner	USA	Liquid	Electrochemical
Global Thermostat	USA	Solid	Temperature and vacuum
GreenCap Solutions	Norway	Solid	Temperature
Greenlyte Carbon Technologies	Germany	Liquid	Electrochemical
Heimdal	USA	Solid	Temperature
Heirloom	USA	Solid	Temperature
Holocene	USA	Liquid	Temperature
Holy Grail	USA	Liquid	Not specified
Homeostasis	USA	Liquid	Electrochemical
Honda	Japan	Not specified	Not specified
Hydrocell	Finland	Solid	Temperature and vacuum
InnoSepra	USA	Solid	Temperature
Ionada	Canada	Membrane	Temperature and vacuum
Jeevan Climate Solutions	USA	Solid	Temperature
Kanata	Canada	Not specified	Temperature
Karbonetiq	USA	Solid	Not specified
Kawasaki	Japan	Solid	Temperature and vacuum
Krajete/Audi	Austria	Solid	Temperature and vacuum
Linhe Climate Science & Technology	China	Solid	Moisture
LowCarbon	South Korea	Not specified	Not specified
Mission Zero	United Kingdom	Liquid	Electrochemical
Mosaic Materials	USA	Solid	Temperature and vacuum
MOVA Technologies	USA	Not specified	Not specified
NEG8 Carbon	Ireland	Solid	Temperature and vacuum
NeoCarbon	German	Solid	Temperature and vacuum
Noya	USA	Solid	Temperature and vacuum
NuAria	USA	Solid	Not specified
Nu̅xsen	USA	Solid	Not specified
OBRIST Group	Austria	Liquid	Electrochemical
Octavia Carbon	Kenya	Solid	Temperature and vacuum
Orbital Materials	United Kingdom	Solid	Temperature
Origen	United Kingdom	Solid	Temperature
Parallel Carbon	USA	Solid	Electrochemical
Phlair	Germany	Liquid	Electrochemical
Planet Savers	Japan	Solid	Temperature and vacuum
Porsche/Volkswagen	Germany	Solid	Temperature
Precision Combustion	USA	Solid	Temperature
Prometheus Fuels	USA	Liquid	Electrochemical
Provocative	USA	Not specified	Not specified
RedoxNRG	Estonia	Membrane	Electrochemical
Removr	Norway	Solid	Temperature
RepAir	Israel	Membrane	Electrochemical
Rivan Industries	United Kingdom	Solid	Temperature
SCW Systems	The Netherlands	Not specified	Not specified
Shell	USA	Solid	Temperature
Sirona Technologies	Belgium	Solid	Temperature and vacuum
Skyrenu	Canada	Solid	Temperature
Skytree	The Netherlands	Solid	Temperature and vacuum
SkyVac	USA	Solid	Not specified
Soletair Power	Finland	Solid	Temperature and vacuum
Sora Fuel	USA	Liquid	Electrochemical
Sosna Metelyk	USA	None	Temperature (cryogenic)
South Ocean Air	USA	Solid	Moisture
Southern Green Gas	Australia	Solid	Temperature and vacuum
SpiralWave	USA	Not specified	Plasma
Spiritus	USA	Solid	Temperature
Stathmos	France	Solid	Temperature
Sustaera	USA	Solid	Temperature
Susteon	USA	Solid	Temperature
Synergetic	USA	Not specified	Not specified
TerraFixing	Canada	Solid	Temperature and vacuum
Terraform Industries	USA	Solid	Temperature
Thalo Laboratories	USA	Solid	Not specified
Travertine	USA	Liquid	Not specified
UAP	United Kingdom	Not specified	Not specified
Ucaneo	Germany	Liquid	Electrochemical
Unemit	USA	Solid	Temperature
UrjanovaC	India	Not specified	Not specified
Valiidun	USA	Not specified	Not specified
Verdox	USA	Membrane	Electrochemical
WindCapture Technologies	Ireland	Solid	Temperature and vacuum
x/44	USA	Liquid	Electrochemical
Yama	France	Liquid	Temperature and Electrochemical
ZeoDAC	USA	Solid	Temperature

## 7 Challenges, Gaps, and Perspectives

The diverse materials considered for the DAC application such as MOFs, zeolites, alkali carbonate-modified carbons, and amine-functionalized silicas offer, at the same time, their distinctive strengths and constraints. Zeolites show robust selectivity for CO_2_ because of their crystalline microporous networks. Nevertheless, they face hindrances from the high temperature of regeneration and moisture competition under humid conditions. At low concentrations of CO_2_, polymers and functionalized silicas show a high uptake of CO_2_ with adjustable selectivity. However, they are susceptible to degradation by the volatilization of amine over long cycles;^330^ besides, their performance depends on the operational temperature.^232^ At low partial pressures, MOFs demonstrate tunable structures and a high affinity for CO_2_. Yet, the energy efficiency and scalability of MOFs are limited due to concerns about their hydrothermal stability. K_2_CO_3_ and Na_2_CO_3_ alkali-modified activated carbons appeared as energy-efficient, low-cost, and relatively stable over the cycles, with moderate CO_2_ uptake values at certain temperature and pressure conditions. Even so, a trade-off stays between adsorption capability, the efficiency of desorption, and the concentration of the CO_2_ output, demanding a specifically designed sorbent to match the specific application requirements. Mostly, the solid sorbents utilized for DAC applications naturally adsorb moisture alongside the adsorption of CO_2_. In various situations, these sorbents demonstrate a higher level of affinity toward humidity than toward CO_2_. Sometimes, the behavior of coadsorption can be beneficial for the process of regeneration. Still, under regular humid conditions, the capacity of the sorbent, selectivity, and stability is reduced. Therefore, recognizing the paired dynamics of water and CO_2_ adsorption experimentally and computationally under an unstable ambient environment is critical.

At this time, DAC technologies are in the stage of growth. Even with the availability of DAC technologies, the adaptation of large-scale technologies faces economic, societal, and political challenges. The shortage of governmental support yields the adaptation of the large-scale technologies challenging. Besides, tackling global warming requires 2.4 million DAC plants. Each plant has a CO_2_ adsorption capacity of 4000 tons. That number needs to be spread over an area equal to 2.5 times that of the New York City area. Currently, 19 DAC plants are operational capturing 0.01 million metric tons.^8^ An enormous amount of funding is necessary for establishing this large number of units across the world. DAC approach selection is entirely crucial in order to cut operational costs and optimize performance. The capabilities of CO_2_ sorption, stability, energy requirements, and technology robustness are required during the selection of suitable DAC methods. Moreover, life cycle assessments alongside the environmental and economic feasibility of DAC technologies must be considered to address the current needs of CO_2_ capture.

Novel materials creation and expenditure-related design are the key focuses of the academic field for the development of new and efficient DAC technologies. As a portion of the ultimate assessment of the sorbent, the analysis of the material structure is necessary. Numerous adsorption configurations such as rotating bed, fluidized bed, and fixed bed can be considered under a wide range of process conditions. The synergetic development of new devices, materials, and structures is expected to overcome the large-scale adoption technical issues. The evaluation of the DAC systems’ effectiveness is based on the key metrics like CO_2_ selectivity of the sorbent, capacity, robustness, energy requirements for regeneration, cyclicality, and performance under diverse conditions of pressure, temperature, and humidity. For the sorbent, mechanical stresses, thermal stability, and long-term stability, in addition to the economic and environmental feasibility, are uniformly critical.

As an outcome of the DAC system distribution, major environmental and social positive impacts could be accomplished. The fertilization of DAC is able to enhance the growth of plants and assist in the alleviation of global hunger via the production of byproducts, food, feed, and other biochemicals. Furthermore, a portable, compact-sized DAC system is able to maintain the air quality in every single living unit, which can also be used as an appliance similar to water purifiers. The DAC systems can be paired with electrochemical conversion cells or dry reforming units for the reduction of carbon to biofuels and the storage of energy. Moreover, the DAC systems can be paired with catalytic converters to convert the captured gas to useful products like gasoline, methanol, and aviation fuels using gas-to-liquid converters.

## 8 Conclusion

The escalating urgency of the global climate crisis has cemented DAC not merely as an optional mitigation strategy, but as an indispensable technological imperative for achieving net-zero emissions by 2050. As demonstrated throughout this review, capturing CO_2_ from ultradilute atmospheric concentrations (∼426 ppm) presents a profound thermodynamic challenge, requiring a delicate equilibrium between binding affinity, sorption kinetics, and the energetic penalties associated with regeneration.

Advances in solid and liquid sorbents have pushed the boundaries of equilibrium CO_2_ uptake capacity. Amine-functionalized mesoporous silicas and metal-exchanged zeolites provide robust baseline performance, while the highly tunable pore architectures of MOFs and POPs offer unprecedented control over selectivity and capture thermodynamics. Furthermore, chemically activated carbon-based materials have emerged as highly competitive, low-cost alternatives with rapid kinetics. However, the paradigm of DAC material design must shift from optimizing the absolute equilibrium capacity toward maximizing the cyclic working capacity under realistic, humid ambient conditions. The ultimate viability of these materials hinges on their resilience against oxidative and thermal degradation over thousands of adsorption–desorption cycles.

Crucially, the translation of high-performance sorbents from the laboratory to the megaton scale requires rigorous co-optimization with process engineering. The design of air contactors, whether gas–solid, gas–liquid, or membrane-based, must drastically minimize pressure drops to reduce parasitic blower loads. Concurrently, advanced regeneration configurations, particularly temperature-vacuum swing adsorption (TVSA) and moisture-swing mechanisms, are critical for circumventing the high thermal energy requirements that currently dominate the operational expenditures of DAC.

Looking forward, the rapid commercialization spearheaded by entities such as Climeworks, Carbon Engineering, and Global Thermostat proves the technological readiness of DAC. Yet, scaling from the current kiloton capacity to the required gigaton threshold necessitates aggressive cost reduction toward a $100/ton CO_2_ target. Future research must prioritize holistic, system-level approaches integrating artificial intelligence for rapid sorbent discovery, conducting rigorous life-cycle assessments, and coupling DAC facilities with renewable energy grids and carbon utilization pathways. Ultimately, bridging the gap between molecular-level thermodynamic efficiency and macro-scale techno-economic viability will dictate the success of DAC in restoring the global carbon balance.
